# SIRT3 Activation a Promise in Drug Development? New Insights into SIRT3 Biology and Its Implications on the Drug Discovery Process

**DOI:** 10.1021/acs.jmedchem.3c01979

**Published:** 2024-01-23

**Authors:** Chiara Lambona, Clemens Zwergel, Sergio Valente, Antonello Mai

**Affiliations:** † Department of Drug Chemistry and Technologies, Sapienza University of Rome, Piazzale Aldo Moro 5, 00185 Rome, Italy; ‡ Pasteur Institute, Cenci-Bolognetti Foundation, Sapienza University of Rome, Piazzale Aldo Moro 5, 00185 Rome, Italy

## Abstract

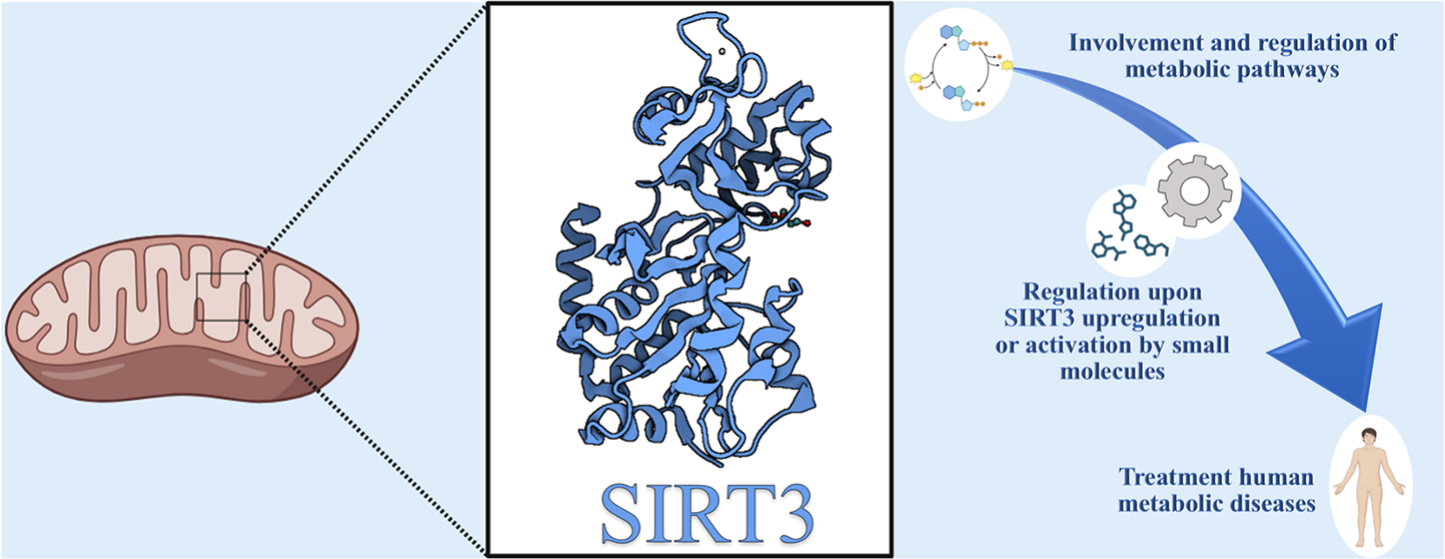

Sirtuins catalyze deacetylation of lysine residues with a NAD^+^-dependent mechanism. In mammals, the sirtuin family is composed of seven members, divided into four subclasses that differ in substrate specificity, subcellular localization, regulation, as well as interactions with other proteins, both within and outside the epigenetic field. Recently, much interest has been growing in SIRT3, which is mainly involved in regulating mitochondrial metabolism. Moreover, SIRT3 seems to be protective in diseases such as age-related, neurodegenerative, liver, kidney, heart, and metabolic ones, as well as in cancer. In most cases, activating SIRT3 could be a promising strategy to tackle these health problems. Here, we summarize the main biological functions, substrates, and interactors of SIRT3, as well as several molecules reported in the literature that are able to modulate SIRT3 activity. Among the activators, some derive from natural products, others from library screening, and others from the classical medicinal chemistry approach.

## Significance

Sirt3 is an epigenetic target from the family sirtuins deacetylases with rapidly growing interest in age-related, neurodegenerative, liver, kidney, heart, and metabolic diseases, as well as in cancer. Its high relevance in numerous diseases is attracting medicinal chemists to develop activators, as Sirt3 activation is associated with beneficial effects to tackle the mentioned health problems. The present perspective is shedding light on the recent findings and potential developments regarding Sirt3 from a medchem viewpoint.

## Introduction

**Figure 1:**
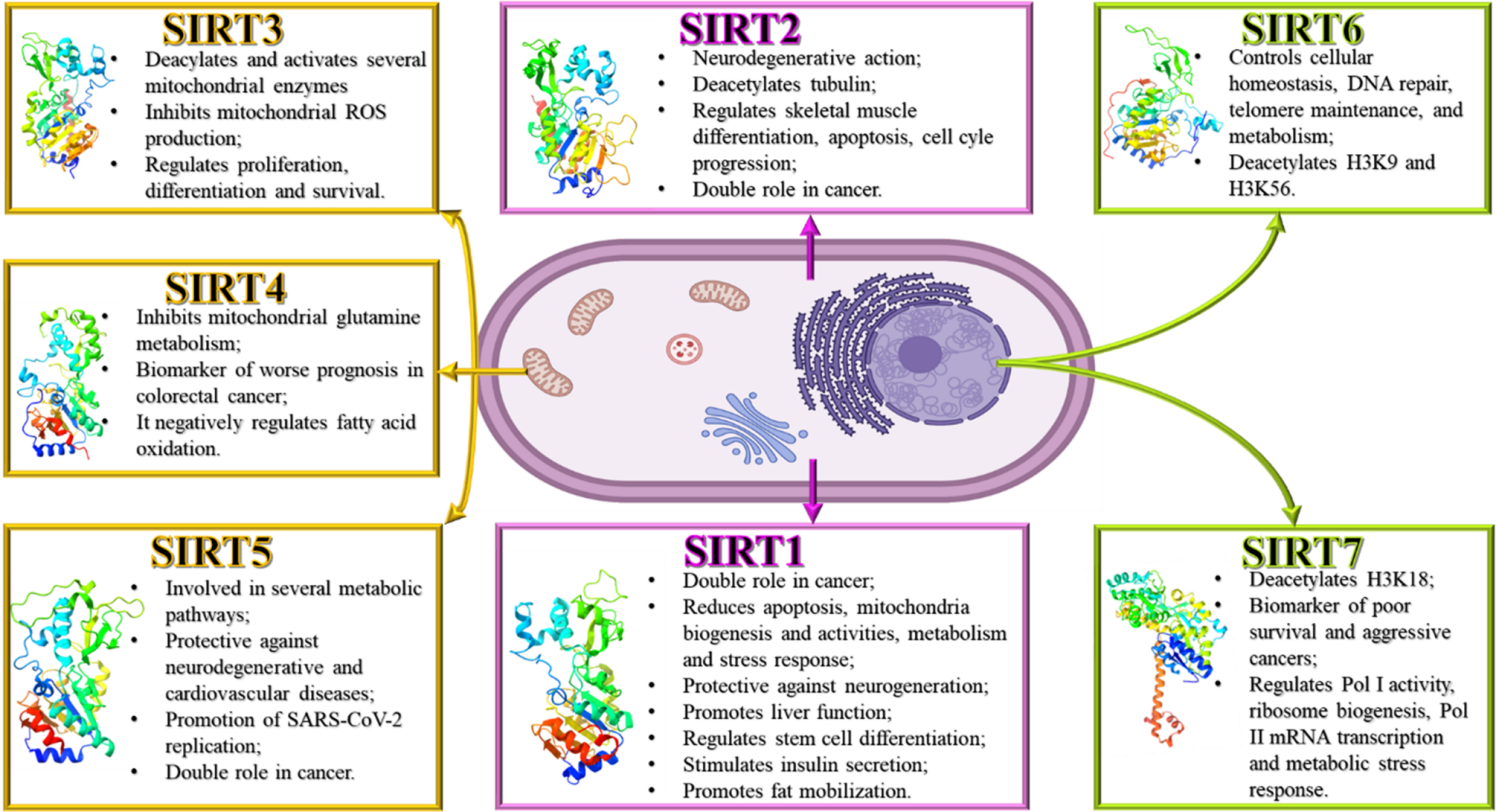
Sirtuin family with its cellular localization and functions (PDB SIRT1, 4IG9; SIRT2, 5D7O; SIRT3, 3GLS; SIRT4, 5OJN; SIRT5, 4F56; SIRT6, 3PKI; SIRT7, 5IQZ).

Histone deacetylases (HDACs) can be classified into two main families: on the one hand, the canonical zinc-dependent histone deacetylases which are subdivided into class I (HDAC1–3, -8), class IIa (HDAC4, -5, -7, -9), class IIb (HDAC6, -10), and class IV (HDAC11),^1^ and on the other hand, the sirtuins (SIRTs) known as class III HDACs.^1−3^ The term “sirtuin” originates from its first discovered member, Sir2, initially identified in *Saccharomyces cerevisiae*, where the acronym SIR stands for silent information regulator.^2^ SIRTs, found in both prokaryotes and eukaryotes, are enzymes that rely on NAD^+^ for their lysine deacetylase activity. In mammals, the sirtuin family contains seven members with different cellular localization and biological roles, whereas in bacteria and archaea, just one or two members were found yet.^4^ SIRTs exhibit a remarkably conserved catalytic core domain composed of 275 amino acids while diverging at their *C*- and *N*-terminal domains in length and sequence in both. Each of the seven human SIRTs prefers an isoform-specific substrate due to very small variations in their substrate binding site, whereas isoform differences in preferred substrate acyls are influenced by the way the acyl moiety binds to an active site channel.^5^ SIRTs differ not only in their substrate specificity but also in their cellular localization, in the binding mode to potential regulatory molecules, and in the protein interactions. In the last two decades, sirtuins attained more and more attention because of their pivotal functions in several biochemical contexts, such as the regulation of cytodifferentiation, transcription, cell cycle progression, inflammation, energetic metabolism, apoptosis, neuro- and cardio-protection, cancer initiation, and progression.^6−8^ Considering their shared conserved catalytic core domain, sirtuins are divided into four subclasses: class I contains SIRT1, -2, and -3, mainly showing deacetylase activity. SIRT1 and SIRT2 can be found in the cytoplasm and nucleus; however, SIRT1 is mainly located in the nucleus, while SIRT2 has predominantly a cytoplasmic localization. SIRT3, apart from its nuclear localization, can also be found in the mitochondria. Class II contains SIRT4 that shows mono-ADP-ribosyltransferase activity in addition to deacetylase and deacylase ones in mitochondria. Class III includes SIRT5, which deacylates succinyl-, glutaryl-, and malonyl-lysine residues in mitochondria. SIRT5′s substrate specificity is given by Tyr102 and Arg105 localized into the substrate pocket. These two residues can form hydrogen bonds and electrostatic interactions with the negatively charged acyl-lysine. Moreover, SIRT5 has an Ala86 to recognize substrates, whereas in the same position, SIRT1–3 have a phenylalanine. The smaller size of alanine with respect to phenylalanine results in a larger binding site that can accommodate larger acylated lysine substrates.^9,10^ According to kinetic studies by Roessler et al., SIRT5 has the best catalytic efficiency for deglutarylation, followed by desuccinylation and then demalonylation.^11^ Class IV is comprised of two members, SIRT6, which shows activities similar to SIRT4 but has a nuclear subcellular localization, and SIRT7 being a nucleolar deacetylase (Figure 1).^4,12^ Among the mammalian sirtuins, a robust deacetylase activity is only shown by members of class I, while SIRT4–7 possess only a very weak deacetylase activity *in vitro.*(1) Sirtuins influence multiple biological processes, such as regulation of metabolism, chromatin biology, transcription, inflammation, cell cycle, apoptosis, autophagy, immune response, oxidative stress, DNA repair, cell differentiation, and microtubule dynamics.^13^

The roles of each sirtuin in human physiology and pathology are different. SIRT1, the best-studied mammalian sirtuin,^1^ seems to have a contradictory role in cancer, acting as a tumor promoter or suppressor. Patients with high expression of SIRT1 develop resistance to chemotherapy more easily than those with low levels.^6^ SIRT1 can reduce apoptosis through p53, regulate mitochondria biogenesis and activities through peroxisome proliferator-activated receptor gamma coactivator 1-alpha (PGC-1α) but also regulate metabolism and stress response via forkhead box O (FOXO) transcription factors deacetylation. Furthermore, SIRT1 can protect against neurodegenerative diseases, promote liver function, regulate stem cell differentiation and cell fate, repress the expression of mitochondrial uncoupling protein 2 (UCP2) in pancreatic β-cells stimulating insulin secretion, and promote fat mobilization by silencing genes controlled by peroxisome proliferator-activated receptor gamma (PPAR-γ).^2^ In prostate cancer, SIRT1 seems to induce epithelial-to-mesenchymal transition (EMT) by suppressing E-cadherin expression.^1^ SIRT1 is associated with epigenetic silencing and heterochromatin formation.^1^ SIRT2 has a neurodegenerative action in neurological diseases,^6^ deacetylates α-tubulin and regulates skeletal muscle differentiation.^2^ Moreover, SIRT2 controls apoptosis through p53 deacetylation and regulates cell cycle progression at many levels. In cancer, SIRT2 is a double-edged sword, acting as a cancer suppressor and promoter.^14,15^ SIRT2 is also involved in metabolic processes.^16^ SIRT3 inhibits mitochondrial reactive oxygen species (ROS) production and regulates proliferation, differentiation, and survival through interaction with different mitochondrial proteins. SIRT4 can arrest the cell cycle by inhibiting mitochondrial glutamine metabolism. The expression of SIRT4 correlates with a worse prognosis in colorectal cancer.^6^ In liver and muscle cells, SIRT4 negatively regulates fatty acid oxidation.^2^ Due to its mitochondrial localization, SIRT5 is engaged in glycolysis, fatty acid and amino acid metabolism, mitochondrial functions, and ROS management. Based on the substrate, SIRT5 can have a promoting or a suppressive action in the metabolic pathway in which the substrate is involved. Considering the role of SIRT5 in noncancer diseases, it seems to have a protective role in neurodegenerative and cardiovascular diseases.^17^ Recently, Walter et al. found that SIRT5 can promote the viral replication of SARS-CoV-2.^18^ In cancer, SIRT5 has a controversial role. SIRT5 seems to act as a tumor suppressor in glioma, gastric cancer, and pancreatic ductal adenocarcinoma (PDAC). Instead, in non-small cell lung cancer (NSCLC), neuroblastoma, ovarian cancer, osteosarcoma, cutaneous and uveal melanoma, and acute myeloid leukemia (AML), SIRT5 seems to act as a tumor promoter. Moreover, SIRT5 plays a dual role as a promoter/suppressor in lung cancer, hepatocellular carcinoma (HCC), breast cancer, and prostate cancer.^17^ SIRT6 controls cellular homeostasis, DNA repair, telomere maintenance, and metabolism.^6^ SIRT6 deacetylates H3K9 and H3K56 to maintain genome stability and telomere function. Furthermore, under oxidative stress, SIRT6 functions as an ADP-ribosylase for poly ADP-ribose polymerase 1 (PARP1).^2,19^ Finally, SIRT7 deacetylates H3K18, a biomarker of aggressive tumors, and elevated expression of SIRT7 is correlated with poor survival rates and increased aggressiveness.^6^ SIRT7 interacts with RNA pol I, rDNA transcription factor UBF, and chromatin remodeling complex WICH.^2^ SIRT7 is able to regulate Pol I activity and ribosome biogenesis, Pol II mRNA transcription and metabolic stress response, and very likely the transcription of Pol III as well (Figure 1).^1,2,6,17,19^ All isoforms share the same catalytic deacylation mechanism because of the homology of their catalytic core. For the deacylation, sirtuins consume NAD^+^. The reaction products are nicotinamide, the deacylated substrate, and *O*-acyl ADP-ribose (O-AADPR). The deacylation reaction can be divided into six steps, as outlined in detail by Nogueiras et al. and Feldman et al.^20,21^

## SIRT3 Structure

**Figure 2:**
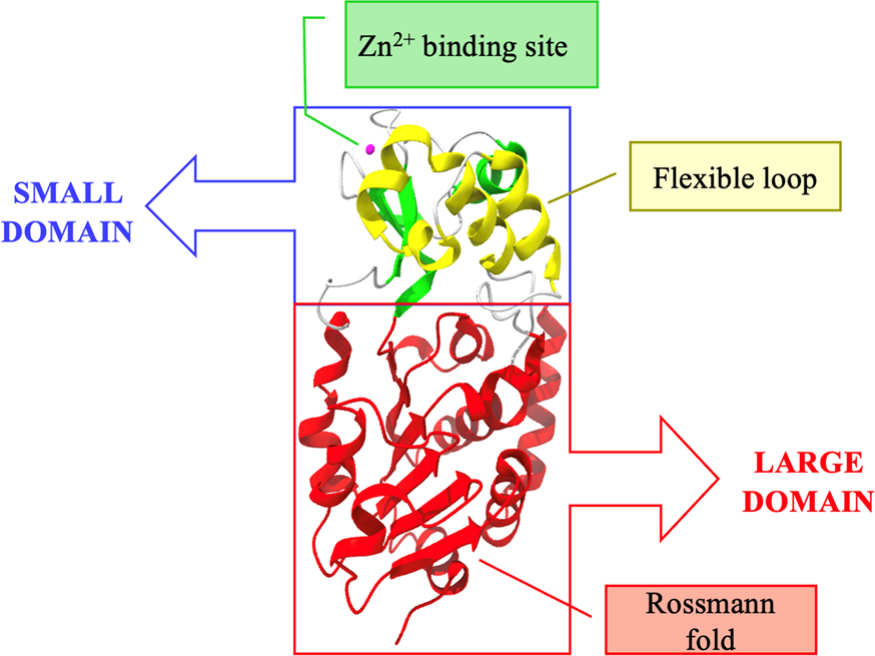
Crystal structure of SIRT3 depicted as a cartoon. The zinc-binding site is highlighted in green, with the zinc ion depicted in pink, the helical module is highlighted in yellow, and the Rossmann fold-like domain is represented in red (PDB 3GLS).

Like the other sirtuins, the structure of the SIRT3 catalytic core consists of two units: a large domain and a small domain. The large domain possesses an inverted Rossmann fold to allow the NAD^+^ binding, while the small domain consists of a helical and a zinc finger module. In the small domain, a flexible loop is present, which changes its conformation during the catalysis. A slender polypeptide chain connects the two domains, while a large groove is formed by three polypeptide chains in the larger domain. Substrates bind to the cleft formed between the two domains. Studies indicate that if these sites mutate, sirtuins lose their catalytic activity (Figure 2).

**Figure 3:**
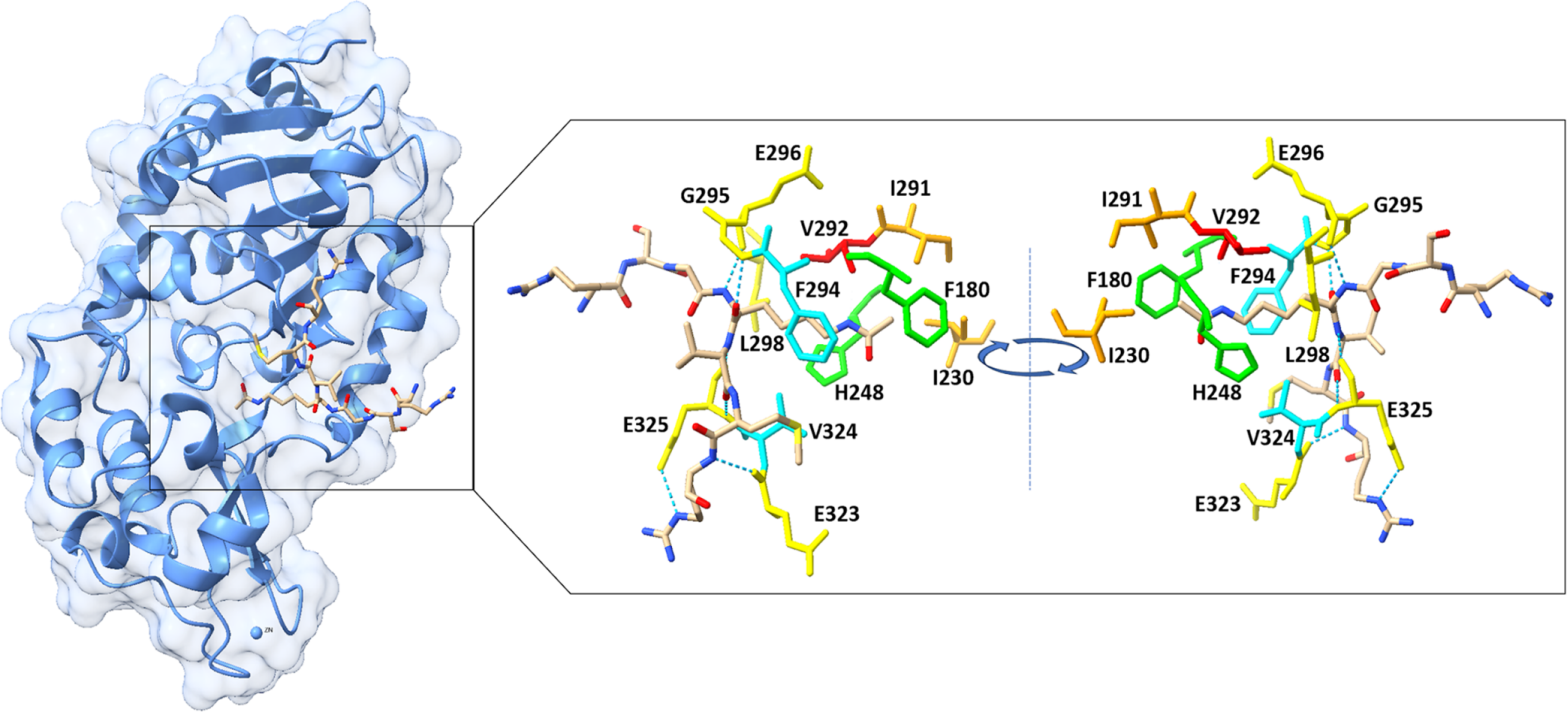
Crystal structure of SIRT3 in complex with AceCS-2 12-mer peptide. (left) The full SIRT3 structure with its substrate analogue. (right) The most important residues for the binding of the substrate. In particular, residues G295, E296, and L298 of the large domain and residues E323 and E325 of the small domain that form hydrogen bonds are depicted in yellow. F294 and V324 are depicted in cyan. H248 and F180 are depicted in green. V292 is highlighted in red. I291 and I230 are depicted in orange. H bonds are depicted with blue dotted lines (PDB 3GLR).

Lei Jin and co-workers^22^ cocrystallized SIRT3 with an acetyl-CoA synthase 2 (AceCS2) peptide of 12 amino acids. The 12-mer peptide contains an AcK642 residue that is deacetylated by SIRT3. The AceCS2 peptide binds the groove between the two domains. In particular, they found that the peptide forms hydrogen bonds with residues G295, E296, and L298 of one loop from the small domain and with residues E323 and E325 from the large domain. F294 and V324 surround the aliphatic portion of the acetyl lysine. These residues are conserved in all sirtuins. H248 and F180 enclose the acetyl group, while the *N*-terminal of the lysine forms a hydrogen bond with the carboxyl of V292. H248 is necessary for the catalytic activity of sirtuins. Indeed, it is conserved among all sirtuins like also I291 and I230. The latter form van der Waals contact with the methyl group on the acetyl lysine. NAD^+^ is necessary for the catalytic activity of SIRT3. In the above-mentioned work, the authors found that the substrate can form a stable complex with SIRT3 in the absence of NAD^+^. They state that NAD^+^ efficiently binds SIRT3 after the binding of the substrate. This suggests that the binding of the substrate in the groove can promote a productive conformation, allowing the binding of NAD^+^. To confirm that, an isothermal titration calorimetry (ITC) study was performed. (Figure 3).^22^

Of note, there is a difference in the binding between SIRT3 and AceCS2-Kac substrate in comparison with SIRT5 and SIRT2 as these sirtuins differ in the flexible loop.^22^

## SIRT3 Involvement in Metabolic Pathways

Much interest is currently growing toward mitochondrial SIRT3, where it is mainly located and plays a vital role in regulating mitochondrial metabolism by influencing the electron transport chain (ETC)/oxidative phosphorylation (OXPHOS), ROS detoxification mechanisms, the tricarboxylic acid (TCA) cycle and urea cycle, amino acid metabolism, fatty acid oxidation, mitochondrial dynamics, and the mitochondrial unfolded protein response (UPR).^4,23,24^ Considering all these important pathways, it is not surprising that SIRT3 has numerous roles in both physiological and pathophysiological processes. Full-length SIRT3 is located in mitochondria, while SIRT3 without the *N*-terminal 142 residues is localized in cytoplasm and nucleus at high expression levels.^25^ The *N*-terminus of SIRT3 has a mitochondrial targeting sequence made of an amphipathic α-helix rich in basic residues. The protein is active when the mitochondrial matrix processing peptidase (MPP) cleaves the first 101 residues *in vitro.*(26,27)

SIRT3 is involved in several mitochondrial oxidative pathways. Mitochondria generate ATP via the OXPHOS pathway involving four protein complexes (I–IV) localized in the inner-mitochondrial membrane. The energy carried by high-energy electrons is harnessed to create a proton gradient through the transport of protons from the mitochondrial matrix to the inner membrane space. Finally, in the complex V or ATP synthase, ATP is formed from ADP. Byproducts of this process are ROS that form when electrons escape complex I or complex III and react with oxygen to the superoxide radical.^28^ SIRT3 potentially has an impact on various stages of the OXPHOS pathway.

SIRT3 is physically associated with complexes I, II, and V and seems pivotal for complexes I and III. When the activity of complexes I–III is reduced, the production of ROS increases, and cellular ATP levels are lowered.^29−32^

Every enzyme can be acetylated in the TCA cycle. Up to now, only the succinate dehydrogenase (SDH) is a recognized substrate of SIRT3. SDH corresponds to the complex II of OXPHOS; thus, SIRT3 is involved in both the TCA cycle and OXPHOS. By modulating SDH activity, SIRT3 can coordinate the oxidation through the TCA cycle and thus substrate delivery to the electron transport chain to avoid the possibility of overcharging the electron transport, resulting in ROS production.^30,31^ Another target of SIRT3 is the isocitrate dehydrogenase 2 (IDH2). IDH2 is an NADP^+^-dependent enzyme able to produce NADPH in mitochondria. NADP^+^ does not function as an electron carrier in the ETC; consequently, IDH2 is not classified as an enzyme involved in the TCA cycle. However, IDH2 has crucial roles in cancer metabolism, leading to a reduction of oxidative stress or to a stimulation of anabolic processes. In hypoxic conditions commonly found in the tumor microenvironment, IDH2 is capable of producing citrate.^33^ The glutathione reductase, which converts oxidized glutathione (GSSG) into reduced glutathione (GSH), uses NADPH for this transformation, while GSH serves as a cofactor for the mitochondrial glutathione peroxidase (GPX) to detoxify ROS. Furthermore, SIRT3 can deacetylate the glutamate dehydrogenase (GDH) that can produce NADPH, very likely contributing to the elevated levels of GSH accessible for GPX.^34^ Yet, the process of neutralizing ROS through the production of NADPH and the activation of manganese superoxide dismutase (SOD2), facilitated by SIRT3, is confined to the mitochondrial matrix and does not enhance antioxidant capabilities in the cell’s nucleus or cytoplasm.^34^ SIRT3 can boost fatty acid oxidation by deacetylating the long-chain acyl-CoA dehydrogenase (LCAD)^35^ and GDH to increase amino acid oxidation. LCAD forms acetyl-CoA, while GDH forms α-ketoglutarate. These two molecules can enter and fuel the TCA cycle.^36^ SIRT3 is involved in various mitochondrial pathways essential for the maintenance of mitochondrial homeostasis in case of nutrient deprivation. SIRT3 removes acetyl groups and activates 3-hydroxy-3-methylglutaryl CoA synthase 2 (HMGCS2), which is the enzyme that controls the rate of production of the ketone body β-hydroxybutyrate.^37^ SIRT3 boosts energy generation from ketone bodies through AceCS2^25,38^ and deacetylases ornithine transcarbamoylase (OTC), an enzyme involved in the urea cycle. The removal of acetyl groups through deacetylation triggers the activation of the urea cycle, facilitating the elimination of ammonia when amino acids undergo catabolic processes that strip them of their carbon components.^34,39^ In the metabolism, SIRT3 promotes the entrance of fatty acids, amino acids, and ketone bodies into the TCA cycle to fuel OXPHOS and to produce energy. Furthermore, SIRT3 stimulates the urea cycle and ketone body synthesis to maintain a balance in metabolic processes.^40^ SIRT3 can also deacetylase the antioxidant enzyme SOD2 in the mitochondrial matrix. Studies demonstrate that SIRT3 deacetylases K53, K68, and K122 of SOD2.^41−45^ Moreover, calorie restriction (CR) induces deacetylation and activation of SOD2, very likely via the upregulation of SIRT3. In particular, Qiu et al.^43^ observed that SIRT3 KO mice fed with a caloric restriction diet for 6 months showed higher oxidative stress and damage than WT mice fed with the same diet. This suggests that the drop in oxidative stress during CR requires SIRT3. Moreover, they studied the cellular ROS modulation by overexpressing SIRT3 in WT and SOD2 KO mouse embryonic fibroblasts (MEFs). They found that the overexpression of SIRT3 in WT MEFs reduced cellular ROS by 40%, while this reduction decreased in SOD2 KO MEFs. Consequently, this indicated that SIRT3 lowers cellular ROS by activating SOD2. In line with this statement, they found that the overexpression of SIRT3 in SOD2 KO MEFs treated with paraquat, a compound that generates superoxide, did not impact the cell viability while overexpressing SIRT3 in WT MEFs, the cell survival increased. Finally, they further demonstrated that SOD2 is deacetylated by SIRT3 in a condition of calorie restriction by using SIRT3 WT and KO mice fed ad libitum or CR diets.^43,46^ Moreover, SIRT3 deacetylates cyclophilin D (CypD), resulting in the opening of the mitochondrial permeability transition pore (mPTP) and thus leading to apoptosis.^34,47^ SIRT3 can trigger autophagy, a prominent cellular self-defense mechanism, by deacetylating the liver kinase B1 (LKB1), thus activating the AMPK-mTOR autophagy pathway.^48^ Notably, deacetylated FOXO3α seems to protect cells from apoptosis via activating a range of autophagy-related genes, such as ULK1, ATG5, and ATG7.^49^

Very recently, Hao et al.^50^ found that SIRT3 is able to remove the lactoyl group at H4K16, and they confirmed this with crystal structural analysis, also applying several chemical probes. As for the histone lysine acetylation, the lactylation stimulates gene transcription by maintaining open the chromatin.^51^ The same authors have also demonstrated that SIRT3 prefers to delactylate *N*-lactyl-d-lysine rather than *N*-lactyl-l-lysine. Particularly, crystal analysis reveals that the lysine hydrocarbon chain is located in a hydrophobic pocket of SIRT3, while the hydroxyl group of lactyl-lysine is stabilized by a hydrogen bond network, including some water molecules.^50^

## Regulation of SIRT3

**Figure 4:**
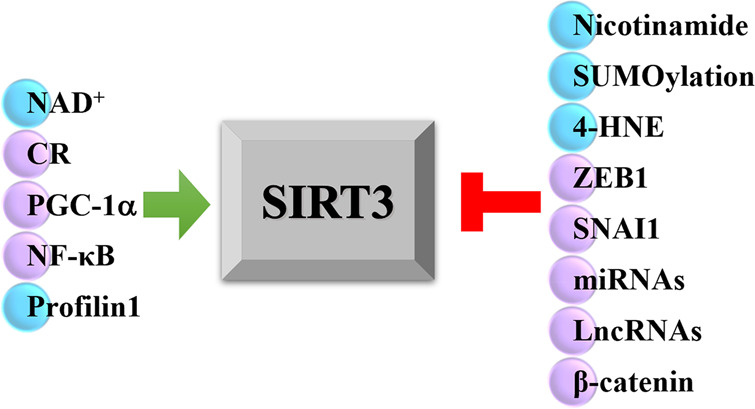
Regulator factors of SIRT3. Factors that exert a direct biochemical effect are indicated with a light blue ball, while the ones that exert a transcriptional effect are indicated with a lilac ball.

As a sensor of mitochondrial energy, SIRT3 has several endogenous regulators that can modulate its expression, such as NAD^+^, its cofactor that stimulates deacetylation-dependent processes, and CR, which lead to increased SIRT3 expression. SIRT3 can be covalently inhibited by 4-hydroxy-nonenale (4-HNE),^52^ while the nuclear factor κB (NF-κB) can bind to the promoter region of SIRT3, leading to an augmentation in its expression.^53^ Similarly, there is some evidence that PGC-1α may stimulate SIRT3 transcription via binding to its promoter,^20,54^ while SNAI1 and Zinc finger Ebox-binding homeobox 1 (ZEB1) exert a negative regulation on the activity of the SIRT3 promoter, effectively suppressing its expression.^55,56^ The byproduct nicotinamide inhibits deacetylation because it binds to the reaction product and accelerates the reverse reaction.^57,58^ SIRT3 can be SUMOylated, and this post-translational modification inhibits its activity. A SUMO-specific protease, SENP1, can de-SUMOylate SIRT3 and thus can promote mitochondrial metabolism.^59^ Moreover, studies demonstrated that several microRNAs can target the 3′UTR of SIRT3, suppressing both gene expression and protein abundance.^60−66^ MicroRNAs are noncoding RNA molecules that can control mRNA stability and protein levels by binding to complementary target mRNA. Of note, miR-210 targets and represses the iron–sulfur cluster assembly protein (ISCU), which changes the NAD^+^/NADH ratio and thus indirectly influences SIRT3.^67^ Furthermore, two long noncoding RNA (lncRNAs) can suppress the mRNA expression of miRNA and thus can positively regulate SIRT3.^65,66^ Finally, SIRT3 can also be regulated by a protein–protein network. Profilin1, an actin-associated protein, can promote the expression of SIRT3,^68^ while β-catenin, an essential downstream mediator within the Wnt signaling cascade, can inhibit the expression of SIRT3 (Figure 4).^4,69^

## SIRT3 and Human Diseases

The SIRT3 protein exhibits a broad distribution in tissues abundant with mitochondria, including but not limited to kidney, heart, brain, and liver tissues,^27^ thus regulating aging, neurodegeneration, and other diseases related to the aforementioned organs (Table 1 and Figures 7,8).^70^ Moreover, SIRT3 is considered a double-edged sword in cancer development.^71^

**Table 1:** Substrates of SIRT3 with Related Pathways and Human Diseases

substrates	pathway/effect	disease	ref
glutamate dehydrogenase (GDH)	amino acid catabolism, NADPH production	metabolic diseases	(36,186,187)
acetyl CoA synthetase 2 (ACS2)	acetate metabolism	liver diseases	(38,188)
ornithine transcarbamoylase (OTC)	amino acid catabolism, urea cycle		(39)
3-hydroxy-3-methylglutaryl CoA synthase 2 (HMGCS2)	ketogenesis	liver diseases	(37)
long-chain acyl-CoA dehydrogenase (LCAD)	β-oxidation	liver diseases, heart diseases	(35)
NADH quinone oxidoreductase (complex I)	OXPHOS	kidney diseases, heart diseases	(29)
succinate dehydrogenase (complex II)	OXPHOS		(30,31,187)
isocitrate dehydrogenase 2 (IDH2)	TCA cycle, NADPH production	hearing loss	(36,130)
manganese superoxide dismutase (MnSOD or SOD2)	redox balance	metabolic diseases, age-related diseases, heart diseases, Alzheimer’s disease, Parkinson’s disease, stroke, TBI	(43,46,164,166,170,182,185)
cyclophilin D (CypD)	apoptosis, glycolysis, improving mitochondrial functions	kidney diseases, age-related diseases, breast cancer, heart diseases, neuropathic pain	(47,91,152)
prolyl hydroxylase domain (PHD)	regulation of HIF-1α activity	breast cancer	(90)
pyruvate dehydrogenase complex (PDC)	inhibition of glycolysis and promotion of apoptosis	lung cancer	(79)
glutamate oxaloacetate transaminase 2 (GOT2)	regulation of glycolysis	pancreatic cancer	(92)
glycogen synthase kinase 3β (GSK3β)	induction of apoptosis	HCC, ameliorates cardiac fibrosis, kidney diseases	(96,123)
p53	induction of apoptosis	HCC, protection of cardiomyocytes, kidney diseases	(93,189)
serine hydroxymethyltransferase 2 (SHMT2)	carcinogenesis	colorectal cancer	(104)
acetyl-CoA carboxylase (ACC1)	promotion of lipid metabolism and cancer migration and invasion	cervical squamous cell carcinoma	(105)
pyrroline-5-carboxylate reductase 1 (PYCR1)	promote cell survival	breast and lung cancer	(106)
ATP5O, ATP5A1	contractile function of the heart	heart diseases	(115)
forkhead box O3a (FOXO3a)	inhibition of mTOR and Rho/Rho-kinase signaling, mitochondrial dynamics	cardiac hypertrophy, kidney diseases, Huntington’s disease, stroke, TBI	(118−120,123,173,174,182,185)
nicotinamide mononucleotide adenylyltransferase 3 (NMNAT3)	-	promotion of antihypertrophic effect	(122)
signal transducer and activator of transcription 3 (STAT3)	inhibition of fibrosis caused by STAT3-NFATc2	cardiac fibrosis	(124)
Ku70	inhibition of apoptosis	heart diseases	(77)
nicotinamide mononucleotide adenylyltransferase 2 (NMNAT2)	interaction with HSP90	age-related diseases	(190)
perossisome proliferator-activated receptor gamma coactivator 1α (PGC-1α)	improving mitochondrial biogenesis and energy generation	kidney diseases	(92)

### SIRT3 and Cancer

**Figure 5:**
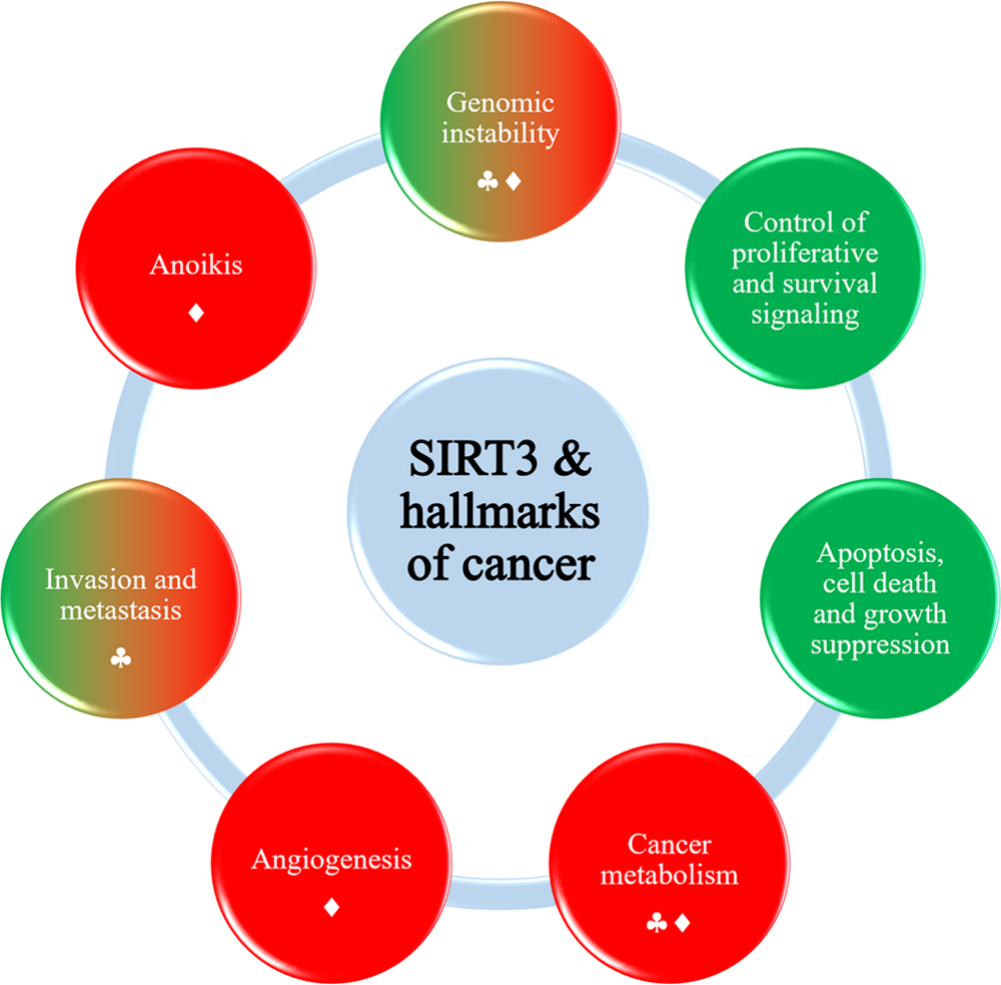
SIRT3 and hallmarks of cancer. Hallmarks promoted by SIRT3 are depicted in green, while those inhibited/downregulated by SIRT3 are indicated in red. The circles with mixed colors green and red represent the hallmarks in which SIRT3 plays a context-dependent role. Inhibition is reported with spades, while downregulation is reported with a rhombus.

SIRT3 plays a role in multiple fundamental features of cancer (Figure 5). SIRT3 may induce genomic instability via its influence on SOD2 and ROS production, given that increased ROS levels have been linked with mutagenesis promotion and genome destabilization.^72^ Moreover, other studies support that under conditions of cellular stress, SIRT3 relocates to the cell nucleus; thus, SIRT3 may have an impact on histone modifications, specifically H4K16Ac and H3K9Ac showing a direct influence of SIRT3 on genomic instability.^73^ SIRT3 can deacetylate H3K59, involved in DNA damage, particularly in enhancing DNA nonhomologous end joining repair.^74^ 8-Oxoguanine-DNA glycosylase 1 (OGG1) is an enzyme that inhibits genome damage, and SIRT3 blocks its degradation to inhibit tumorigenesis.^75^ The function of SIRT3 in controlling the limitless replicative potential of cancer cells is not yet well understood. SIRT3 influences cell proliferation and survival via various mechanisms depending on the cancer type. Nowadays, it is well established that ROS levels are generally lower in normal cells compared to cancerous ones. For example, in cancers overexpressing SIRT3, like head and neck squamous cell carcinoma (HNSCC), SIRT3 keeps the ROS species at an adequate rather low level, resulting in a malignant phenotype.^76^ In HeLa cells, SIRT3 removes acetyl groups from Ku70, leading to increased Ku70-Bax interactions and subsequently facilitating the movement of Bax to the mitochondria, thus leading to cancer cell survival through protection from genotoxic and oxidative stress.^77^

Hypoxia-inducible factors (HIFs) can directly activate the transcription of genes associated with the onset of cancer, such as the vascular endothelial growth factor (VEGF) and the transforming growth factor α (TGF-α). Under hypoxic conditions, overexpression of SIRT3 leads to lower levels of ROS production, decreased glycolysis and proliferation, as well as reduced HIF-1α stabilization, blocking its downstream transcriptional activity, ultimately leading to impaired tumorigenesis.^73,78^ Moreover, SIRT3 can deacetylate the pyruvate dehydrogenase complex (PDC) related to glycolysis. When PDC is deacetylated, glycolysis is inhibited, and apoptosis in cancer cells is promoted.^79^ SIRT3 is also capable of engaging in pathways that lead to apoptotic cell death and the inhibition of cell growth, such as JNK, Bax, HIF-1α, but also ROS. SIRT3 modulates the JNK2 signaling pathway in numerous tumor types, such as osteosarcomas, colorectal carcinoma, but also in healthy human cell lines (lung and retinal epithelial cells), resulting in enhanced growth arrest and apoptosis.^80^ For the involvement of SIRT3 in cancer invasion and metastasis, one study reports that SIRT3 transcription levels are linked with lymph node-positive metastatic breast cancer.^81^ SIRT3 is also involved in anoikis, which represents apoptosis triggered by the dysfunctional cell adhesion to the extracellular matrix (ECM).^82^ Anoikis resistance is correlated to the initiation and progression of cancer via the invasion of neighboring lymph nodes as well as other tissues and organs.^73^ In oral cancer, SIRT3 was demonstrated to mediate anoikis resistance as it is downregulated by the receptor-interacting protein (RIP), which is a known kinase shuttling between Fas-mediated cell death and integrin/focal adhesion kinase (FAK)-mediated survival.^83^

In the cancer context, SIRT3 exhibits a role that varies depending on the specific circumstances, acting as a promoter of tumorigenesis in some types of cancer while acting as a tumor suppressor in others.^84^ Finley et al.^85^ have demonstrated that SIRT3 is additionally involved in controlling glycolytic metabolism by controlling the inducible factor-1α (HIF-1α) stability and activity. This action is closely related to the “Warburg effect” in cancer. The Warburg effect is a hallmark of cancer and indicates the process by which cancer cells use glycolysis even in aerobic conditions, and this confers advantages for tumor growth. HIF-1α is a heterodimer of two basic helix–loop–helix/PAS proteins, HIF-1α, and the aryl hydrocarbon nuclear trans-locator (ARNT).^86^ HIF-1α is unstable at normal oxygen levels (5–21% O_2_), while in low oxygen conditions (0.5–5% O_2_), the HIF-1α subunit becomes more stable, forms a dimer with ARNT, and moves to the cell nucleus, where it binds to the HIF response element (HRE). Transcription mediated by HIF-1α is associated with tumorigenesis because it activates the transcription of genes involved in glucose metabolism, angiogenesis, and metastasis, such as GAPDH, VEGF, and TGF-α.^87^ Abnormal stabilization/activation of HIF-1α is linked to various forms of cancer.^88^ The stability of HIF-1α is increased through the hydroxylation of proline residues within the oxygen-dependent degradation by specific enzymes. In low oxygen conditions, HIF-1α is stabilized by inhibiting the proline hydroxylation enzymes through ROS generated from the complex III of ETC.^89^ SIRT3 controls the HIF-1α activity by direct deacetylation and activating prolyl hydroxylase domain (PHD). As a result, this leads to the tagging of HIF-1α with ubiquitin and its subsequent degradation through the proteasome.^90^ SIRT3 overexpression clearly and reproducibly led to the reduction of stabilized HIF-1α in hypoxic human breast cancer cells. It is noteworthy that SIRT3’s enzymatic function was crucial for the effective repression of HIF-1α target genes because mutated SIRT3 could not significantly reduce hypoxic GLUT1 expression.^85^ SIRT3 may act as an oncosuppressor by regulating glycolytic and anabolic metabolism, as the loss of SIRT3 in human cancer cells is associated with glycolytic gene expression.^40^ SIRT3 functions as the primary deacetylase for the pyruvate dehydrogenase complex (PDC), deacetylating, and activating PDC. This activation inhibits glycolysis and fosters apoptosis in cancer cells.^79^ Moreover, SIRT3 deacetylates and inactivates CypD, inhibiting breast carcinoma glycolysis.^91^ Glutamate oxaloacetate transaminase 2 (GOT2), an enzyme that plays a pivotal role in controlling the glycolysis pathway, is deacetylated and inhibited by SIRT3, hindering pancreatic tumor growth.^92^ Furthermore, SIRT3 can inhibit ROS-modulated tumorigenesis and metastasis.^93^ A decrease in ROS levels by SIRT3 was also proven to attenuate lung adenocarcinoma cell growth.^94^ In chronic lymphocytic leukemia (CLL), SIRT3 activates SOD2 followed by ROS elimination, resulting in inhibited CLL progression.^95^ Additionally, it has been proven that SIRT3 induces programmed cell death by activating, for instance, the glycogen synthase kinase 3β (GSK3β), thus promoting the Bax-regulated apoptosis pathway.^96^ Upregulated SOD2 and p53 activity through SIRT3 further increased Bax- and Fas-regulated apoptosis in HCC.^97^

SIRT3 also seems to be involved in ferroptosis, a cell death mechanism different from the metabolism-independent ones, such as cell apoptosis and necrosis.^98,99^ Ferroptosis is driven by iron-dependent membrane lipid metabolism dysfunction^100^ and can be considered as a tumor suppression mechanism.^101^ Liu et al.^102^ demonstrated that SIRT3 can induce ferroptosis in gallbladder cancer (GBC). Moreover, these researchers also described that SIRT3 can participate in the epithelial-to-mesenchymal transition in GBC. Protein kinase B (AKT), when phosphorylated on Ser473, controls cell survival, migration, and invasion.^103^ They also observed that increased pAKT levels in cells where the SIRT3 gene was knocked down led to the proliferation, migration, and invasion of GBC colonies. Recent evidence has shown that SIRT3 seems to act as a tumor suppressor in GBC.^102^ In colorectal cancer, SIRT3 has the potential to enhance the development of cancer by deacetylating serine hydroxymethyltransferase 2 (SHMT2), thus inhibiting its lysosome-dependent degradation.^104^ SIRT3 can deacetylate acetyl-CoA carboxylase (ACC1) to enhance lipid metabolism, but in cervical cancer cells, this deacetylation promotes cancer migration and invasion.^105^ Another substrate of SIRT3 is pyrroline-5-carboxylate reductase 1 (PYCR1), which correlates with breast and lung cancer cell survival.^106^

### SIRT3 and Liver Diseases

In human and mouse nonalcoholic fatty liver disease (NAFLD) models, SIRT3 is downregulated.^107^ NAFLD is strictly linked with metabolic disorders that involve mitochondrial dysfunctions. Indeed, the name NAFLD has been changed to metabolic dysfunction-associated fatty liver disease (MAFLD) to consider this aspect. It was noticed that exposing SIRT3-deficient mice to a high fat diet (HFD) increases the acetylation of hepatic proteins, reduces the activity of complex III and IV of the ETC, and embitters oxidative stress.^108,109^

In SIRT3-deficient mice, the presence of a high-fat diet worsens conditions such as obesity, insulin resistance, high lipid levels, liver fat accumulation, and inflammation. Nevertheless, when an adenovirus is used to increase SIRT3 expression, it reverses these negative effects.^109^

Moreover, hepatic steatosis is exacerbated by hepatic SIRT3 deficiency in HFD mice, thus leading to an overexpression of protein engaged in the uptake of fatty acids, such as CD36 and VLDL receptors.^110,111^

### SIRT3 and Infectious Diseases

SIRT3 also has a role in infectious diseases such as hepatitis B. SIRT3 is involved in covalently closed circular DNA (cccDNA) transcription and particularly has an antiviral activity through epigenetic regulation. SIRT3 inhibits hepatitis B virus (HBV) RNA transcription without boosting its RNA degradation. The HBV cccDNA is structured like a small chromosome, with a combination of histone and nonhistone proteins helping to organize it; thus, the deacetylase activity of SIRT3 can modulate cccDNA chromatin. Indeed, several studies described an epigenetic control of cccDNA. Particularly, SIRT3 deacetylases H3K9 of HBV cccDNA, and this modification inhibits HBV transcription. Moreover, SIRT3 causes the recruitment of SUV38H1 and SETD1A, two histone methyltransferases, to cccDNA.^112^ Of note, a recent single-center retrospective analysis shows the clinical importance of SIRT3 in individuals affected by COVID-19. It appears that the levels of SIRT3 in the bloodstream are linked to the clinical prognosis and outcome of individuals with COVID-19. In particular, low levels of SIRT3 can be a marker of a severe disease caused by COVID-19.^113^

### SIRT3 and Heart Diseases

SIRT3 participates in several heart diseases, such as heart failure, cardiac hypertrophy, atherosclerosis, and dilated hypertrophy. The contractile function of the heart is impaired by loss of SIRT3.^114^ SIRT3 increases the energy production of mitochondria by activating ATP5O and ATP5A1, two mitochondrial ATP synthases,^115,116^ and the LKB1–AMPK pathway.^117^ Cardiac hypertrophy is an adaptive reaction of the heart to different conditions, and the three crucial pathways involved are the Rho/Rho-kinase one, the mTOR one, and the ROS one. SIRT3 can inhibit cardiac hypertrophy by inhibiting mTOR signaling via activating the LKB1–AMPK pathway.^117^ Moreover, SIRT3 can inhibit mTOR signaling and Rho/Rho-kinase signaling by deacetylating FOXO3a.^118,119^ ROS signaling can be inhibited by deacetylating SOD2.^120^ Another cardiac problem is hypertrophy-related lipid accumulation. SIRT3 can restore lipid metabolism homeostasis by downregulating the acetylation of LCAD.^121^ Moreover, nicotinamide mononucleotide adenylyltransferase 3 (NMNAT3) can promote the antihypertrophic effects of SIRT3 by binding SIRT3 and being deacetylated.^122^ SIRT3 can also ameliorate cardiac fibrosis by deacetylating GSK3β to foster its activity. Active GSK3β can resist the TGF-β/Smad3 pathway that causes cardiac fibrosis.^123^ Furthermore, SIRT3 can deacetylate STAT3 to inhibit the fibrosis caused by STAT3-NFATc2.^124^ Moreover, SIRT3 has a role in the inhibition of autophagy and apoptosis in heart diseases. For instance, when SIRT3 deacetylases Ku70, it interacts with Bax and inhibits the apoptosis of cardiomyocytes.^77^

### SIRT3 and Age-Related Diseases

The SIRT3 activation and the following apoptosis inhibition were shown to improve age-related diseases.^4^ SIRT3 activation or overexpression can potentially hamper age-related macular degeneration and reduce the negative effects of apoptosis,^125^ but also slow down ovarian aging by enhancing mitochondrial function and thus resulting in reduced apoptosis.^126^ Furthermore, SIRT3 can trigger the commencement and activation of the PINK1–Parkin mitophagy pathway, thereby preventing cell demise.^127^ Mitophagy occurs when it is not possible to repair dysfunctional mitochondria.^128^ Nicotinamide mononucleotide adenylyltransferase 2 (NMNAT2) is a substrate of SIRT3 and possesses neuroprotective properties via heat shock protein 90 (HSP90)-interaction, thus resulting in a refolding of aggregated protein substrates. This finding suggests a function of SIRT3 in age-related diseases correlated with the aggregation of pathological proteins such as amyloid beta (Aβ), tau in Alzheimer’s disease (AD), and α-synuclein in Parkinson’s disease (PD). Zhang et al.^48^ reported a protective role of SIRT3 in a rotenone-induced PD cell model through autophagy induction by enhancing the LKB1–AMPK–mTOR pathway.^48^ Aging in inner ear cochlea neurons induces ROS damage in these cells. In CR conditions, SIRT3 has been shown to inhibit ROS-induced damage to cochlea neurons, delaying hearing loss. This action is provided by the SIRT3-mediated deacetylation and activation of IDH2.^129,130^

### SIRT3 and Kidney Diseases

SIRT3 can safeguard the kidneys against metabolic disorders through various mechanisms. Enhanced kidney function is achieved by overexpressing SIRT3, which reduces oxidative damage, mitigates inflammation, and inhibits the apoptosis of renal tubular epithelial cells.^131^ Acute kidney injury (AKI) is a condition with a decrease in glomerular filtration rate characterized by an increase in serum creatinine concentration or oliguria.^132^ In this way, SIRT3 can improve mitochondrial biogenesis and energy generation to protect from AKI. In AKI, SIRT3 can have a protective effect by decreasing the acetylation of CypD and p53.^133,134^ The deacetylation of the latter blocks apoptosis in AKI.^135^ Moreover, SIRT3 can safeguard from AKI by deacetylating PGC-1α and mitochondrial complex I.^136^ Nephrolithiasis, a metabolic kidney disorder, primarily results from damage to renal epithelial cells caused by calcium oxalate. SIRT3 can have some protective role in this disease; indeed, it seems that the SIRT3 expression in nephrolithiasis-afflicted mice is reduced. SIRT3 may inhibit the formation of kidney stones by regulating the erythroid 2-related factor (NRF2)/hemoxygenase 1 (HO-1) pathway.^137^ Renal fibrosis can derive from chronic kidney diseases (CKD).^138^ SIRT3 can inhibit this pathological process both by activating FOXO3a and deacetylating and then activating GSK3β.^123^

### SIRT3 and Metabolic Diseases

SIRT3 is also involved in obesity and diabetes. Insulin resistance and vascular dysfunction are often observed in obese patients in concomitance with SIRT3 deficiency.^108,139^ SIRT3 is able to preserve endothelial cells from mitochondrial ROS damage and can lead to increased nitric oxide (NO) release, resulting in improved vasodilatation.^108^ SIRT3 plays a crucial role in skeletal muscle metabolism and has been proven to activate insulin signaling, thus improving the diabetic condition.^4^ Moreover, SIRT3 maintains bone metabolism by boosting the AMPK-PGC-1β axis.^140^ SIRT3 can inhibit the expression of Ang-2 to retain vascular integrity.^141^ SIRT3 is vital to suppress osteoarthritis^142^ and to decrease atherosclerosis risk.^143^

### SIRT3 and Neuropathic Pain

**Figure 6:**
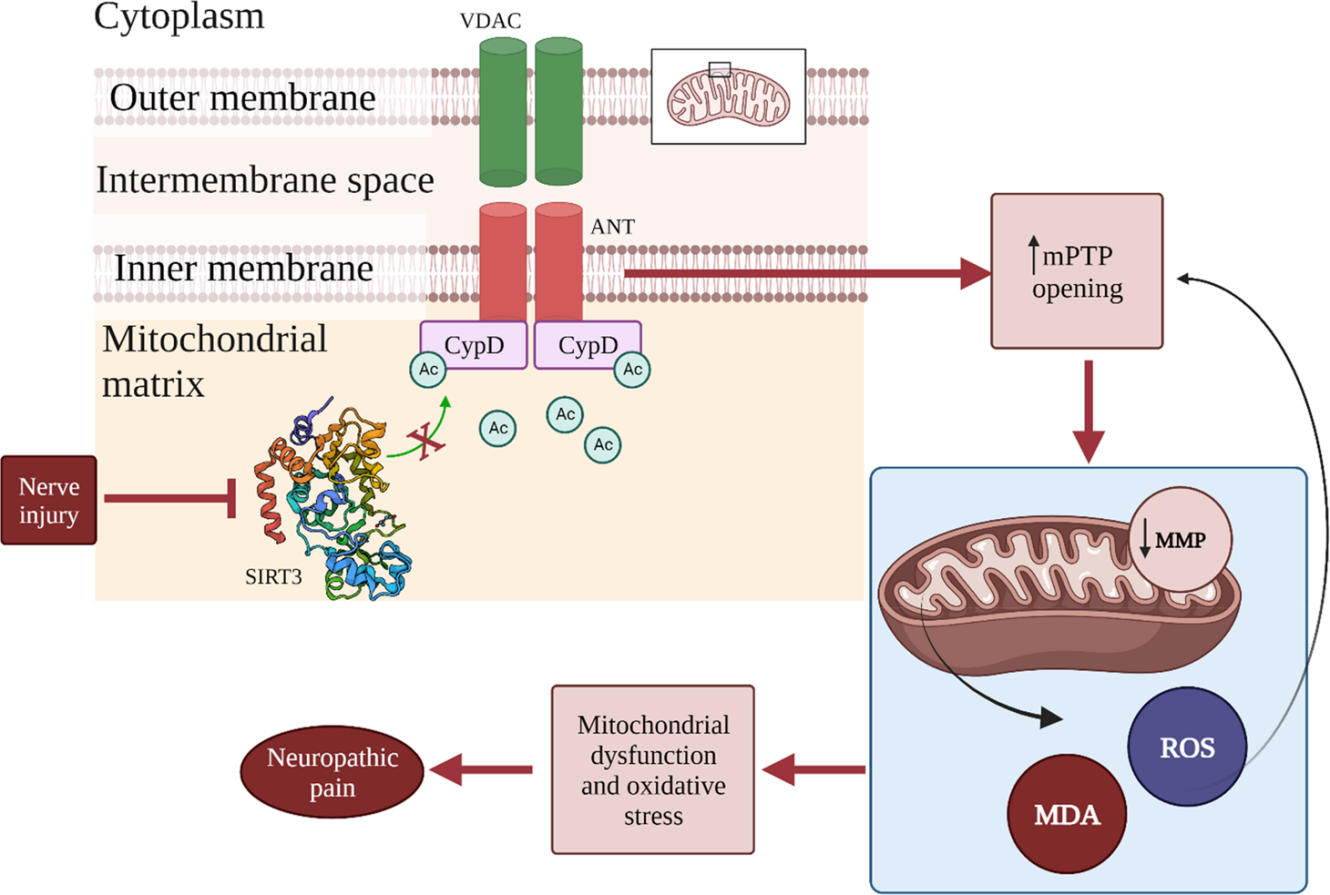
Role of SIRT3 in the development of neuropathic pain. Nerve injury leads to the loss of SIRT3 deacetylase activity on CypD. The red arrows show the consequences of this: increased opening of mPTP, decreased MMP, release of ROS, and MDA. The increase of ROS levels also leads to an augmented opening of mPTP. This contributes to mitochondrial dysfunction and oxidative stress and, finally, participates in the development of neuropathic pain. Adapted with permission from ref (152). Copyright 2022 Hindawi.

Neuropathic pain is a condition that impacts approximately 7–8% of the population in Europe, and at the moment, its etiology is not well-known. Neuropathic pain is a persistent pain condition triggered by damage or disease affecting the somatosensory nervous system. The features of neuropathic pain are spontaneous pain, hyperalgesia, abnormal pain, and paresthesia. Several studies report that mitochondrial dysfunction and oxidative stress can participate in the advancement of neuropathic pain.^144−146^ As previously stated, mitochondria are the main target and source of ROS. In particular, the augmented ROS levels lead to the opening of the mPTP. This can provide a decrease in mitochondrial membrane potential (MMP) and an increased ROS production, thus aggravating mitochondria dysfunction.^146,147^ Some studies link mitochondrial dysfunction with neuropathic pain, suggesting that enhancing mitochondrial function and reducing oxidative stress may represent a promising therapeutic target for addressing this condition. For example, the intraperitoneal injection of cyclosporin A, a specific inhibitor of mPTP, in animals with neuropathic pain can alleviate allodynia and hyperalgesia.^148^ Other researchers propose that administering *tert*-butyl hydroperoxide (*t*-BOOH) via intrathecal injection in typical mice induces pain-related behaviors.^149,150^ Moreover, free radical scavengers and antioxidants can alleviate pain-related behavior in animals suffering from neuropathic pain.^151^ SIRT3 can be involved in this condition because of its deacetylase action on K166-CypD. Yan et al.^152^ have studied this correlation, and their main discovery was that in the spinal cords of mice with the SNI model, there is a decrease in the expression of SIRT3, which leads to an increase in Ac-CypD-K166 levels. By overexpressing SIRT3 in the spinal cord, the pain hypersensitivity in SNI model mice was reversed, and consequently, the acetylation level of K166-CypD was reduced. Moreover, they found that CypD deacetylation reduces allodynia and hyperalgesia in the SNI model mice by enhancing mitochondrial dysfunction and curbing oxidative stress. To affirm the correlation between mitochondrial dysfunction and neuropathic pain, they found that the injection of cyclosporin A protects mitochondrial function and improved pain-like behaviors in mice.^152^ Additionally, the scientists discovered that SIRT3 plays a role in modulating the progression of neuropathic pain within the spinal cord by alleviating mitochondrial dysfunction and suppressing oxidative stress. This finding was confirmed by the evaluation of mPTP, MMP, ROS, and MDA in the spinal cords of SNI mice. In particular, protein and mRNA levels of SIRT3 were reduced, mPTP opening and ROS levels were augmented, MMP was reduced, and MDA were upregulated. All these effects were reversed by overexpressing spinal SIRT3. Moreover, they reported that the involvement of SIRT3 in neuropathic pain is related to its activity on CypD. Indeed, they demonstrated that the acetylation level of CypD was elevated in SNI model mice, and the overexpression of SIRT3 significantly lowered the acetylation level of CypD. CypD can alter the conformation of ANT, a component of mPTP, through acetylation.^153−156^ Moreover, the comparison of wild-type and CypD-K166R mutant SNI model mice indicated that SIRT3 in the spinal cord mediates the deacetylation of CypD, potentially blocking the opening of the mPTP, enhancing mitochondrial resistance to oxidative stress and consequently reducing heightened pain sensitivity in SNI mice. The important role of mPTP in the release of ROS in the cytoplasm,^157−160^ and its involvement in various neurodegenerative diseases^161^ can suggest that mPTP is a crucial regulator in neuropathic pain. Furthermore, given that CypD is an important regulator of mPTP and that SIRT3 can modulate CypD through Ac-K166 deacetylation, it can be stated that SIRT3 represents a prospective promising target for drug development toward neuropathic pain (Figure 6).

### SIRT3 and Neurodegenerative Diseases

Various researches reported that SIRT3 is involved in different neurodegenerative diseases such as AD, PD, Huntington’s disease (HD), neuronal excitotoxicity, stroke, and traumatic brain injury (TBI).^162^

#### AD

It appears that SIRT3 is involved in safeguarding neural integrity, as evidenced by its notable neuroprotective attributes. A decline in SIRT3 expression has been associated with advanced stages of AD, highlighting its potential significance in disease progression.^163^ Furthermore, a noteworthy observation has emerged: upregulating SIRT3 expression holds promise in mitigating certain pathological processes attributed to AD.^164^ Amid the multifaceted effects stemming from augmented SIRT3 expression, a notable outcome involves the deacetylation of SOD2. This event holds the potential to ameliorate mitochondrial processes and bolster homeostasis. In addition, this increased SIRT3 expression has demonstrated the capacity to hamper Aβ-induced PC12 cell death, as well as that induced by H_2_O_2_.^165^

#### PD

**Figure 7:**
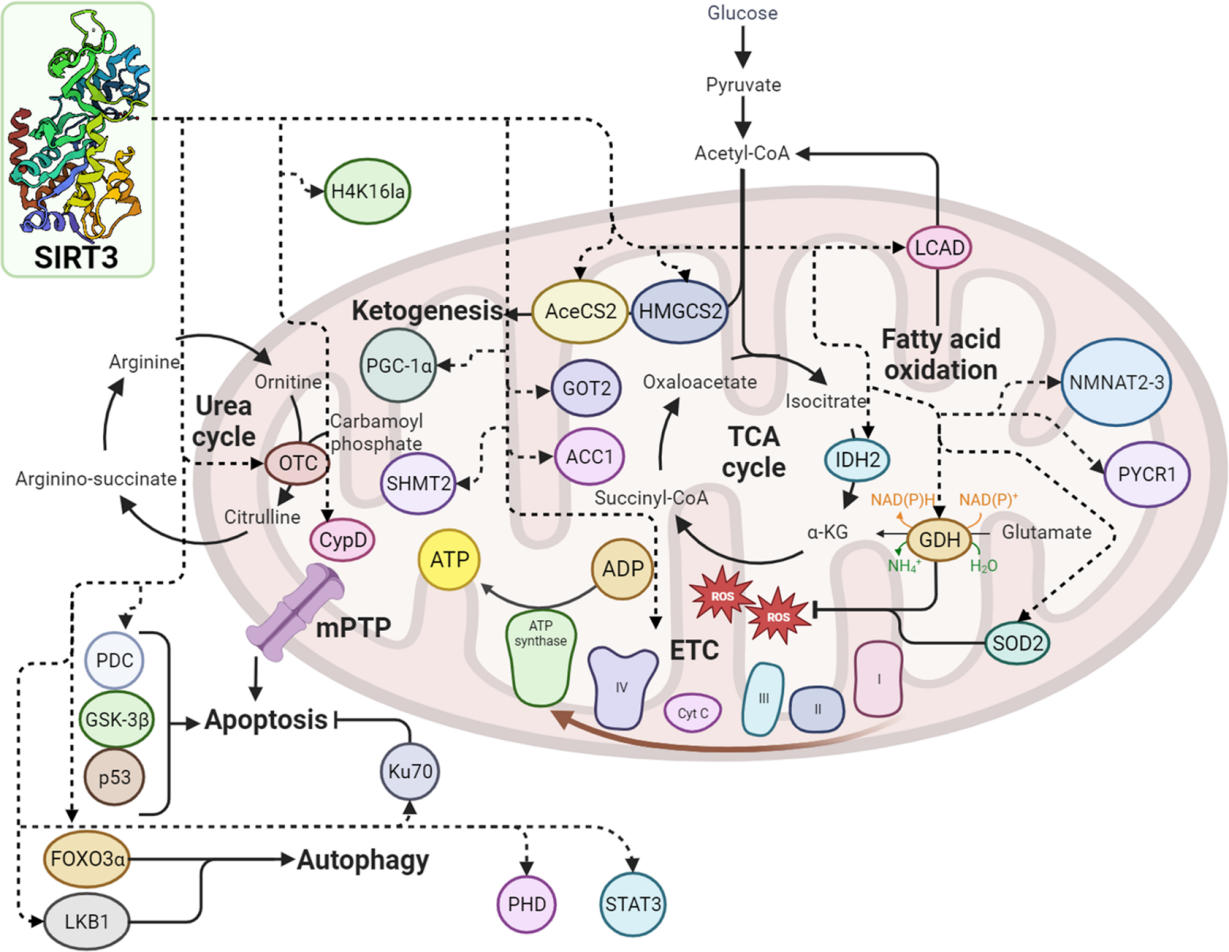
Metabolic pathways involving SIRT3. The dashed arrows indicate the interaction between SIRT3 and other protein factors.

Multiple lines of evidence substantiate the notion that SIRT3 is engaged in the development of PD. Shi et al.^166^ have presented compelling findings showcasing that the absence of SIRT3 results in a diminution of SOD2’s functional capacity. This is attributed to the augmentation of acetylation on K68 of SOD2, culminating in elevated oxidative stress levels and the perturbation of mitochondrial membrane potential. Furthermore, the increased SIRT3 levels have been implicated in the improvement of pathologies induced by 1-methyl-4-phenyl-1,2,3,6-tetrahydropyridine (MPTP) or rotenone, both associated with PD.^167,168^ Elevated expression of SIRT3 serves to counteract the loss of dopaminergic neurons caused by α-synuclein, a protein linked to PD pathology (Figure 7). This protective effect is realized through enhancements in SOD2 functionality and elevations in GSH levels.^169,170^ Conversely, diminished SIRT3 levels within SH-SY5Y cells have been shown to increase both α-synuclein aggregation prompted by rotenone and subsequent cell death.^170^

#### HD

It has been reported that cells expressing mutant huntingtin (mHtt) have a lower expression of SIRT3.^171,172^ mHtt interferes with several mitochondrial processes, such as ETC, ROS generation, and mitochondrial dynamics. SIRT3 is able to restore several of these processes, especially the mitochondrial dynamics, by directly deacetylating optic atrophy 1 (OPA1) and by improving mitofusin-2 (Mfn2) expression through the deacetylation of FOXO3a.^173,174^ OPA1 and Mfn2 are two proteins that increase mitochondrial fission and decrease mitochondrial fusion.

#### TBI

Mitochondrial dysfunctions play a pivotal role in the genesis of stroke, thereby positioning SIRT3 as a compelling candidate for a potential pharmacological intervention.^175−178^ Notably, the manipulation of SIRT3 activity or expression has been shown to yield a reduction in infarct volume.^179^ This reduction in ischemic damage is closely linked with the attenuation of excitotoxicity and the restoration of optimal mitochondrial function.^180,181^ The neuroprotective outcomes observed in the stroke context are intricately tied to the interplay between SIRT3 and the FOXO3a/SOD2 pathway, as well as the intricate dynamics within complex I of the ETC.^182^ In the realm of TBI, SIRT3 involvement has been documented to yield notable improvements in neurobehavioral outcomes within animal models, particularly in the domains of sensorimotor function, learning, and memory.^183−185^ This beneficial impact is attributed to SIRT3’s ability to deacetylate FOXO3a, thereby activating pivotal antioxidant genes such as SOD2 and catalase.^185^ Consequently, a cascading effect ensues, culminating in the mitigation of oxidative stress.^184,185^

## SIRT3 Positive Modulators

Different activators of SIRT3 were described in the literature, able to stimulate SIRT3 expression or biochemical activity (Table 2). Most of the SIRT3 activators reported so far derive from natural products. However, given the recent interest in SIRT3 activation, some novel and synthetic SIRT3 activators are being developed through a medicinal chemistry approach.

**Table 2:**
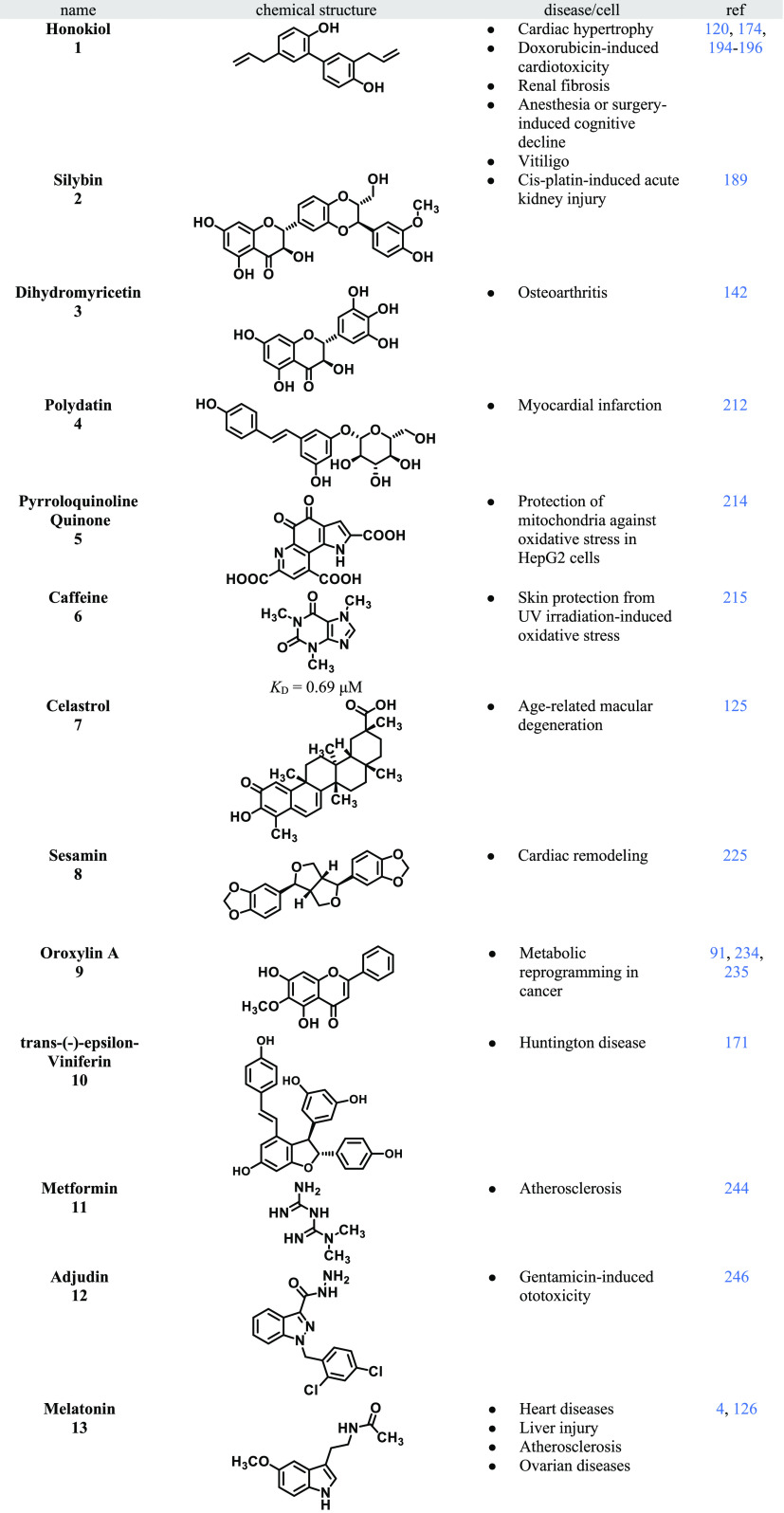

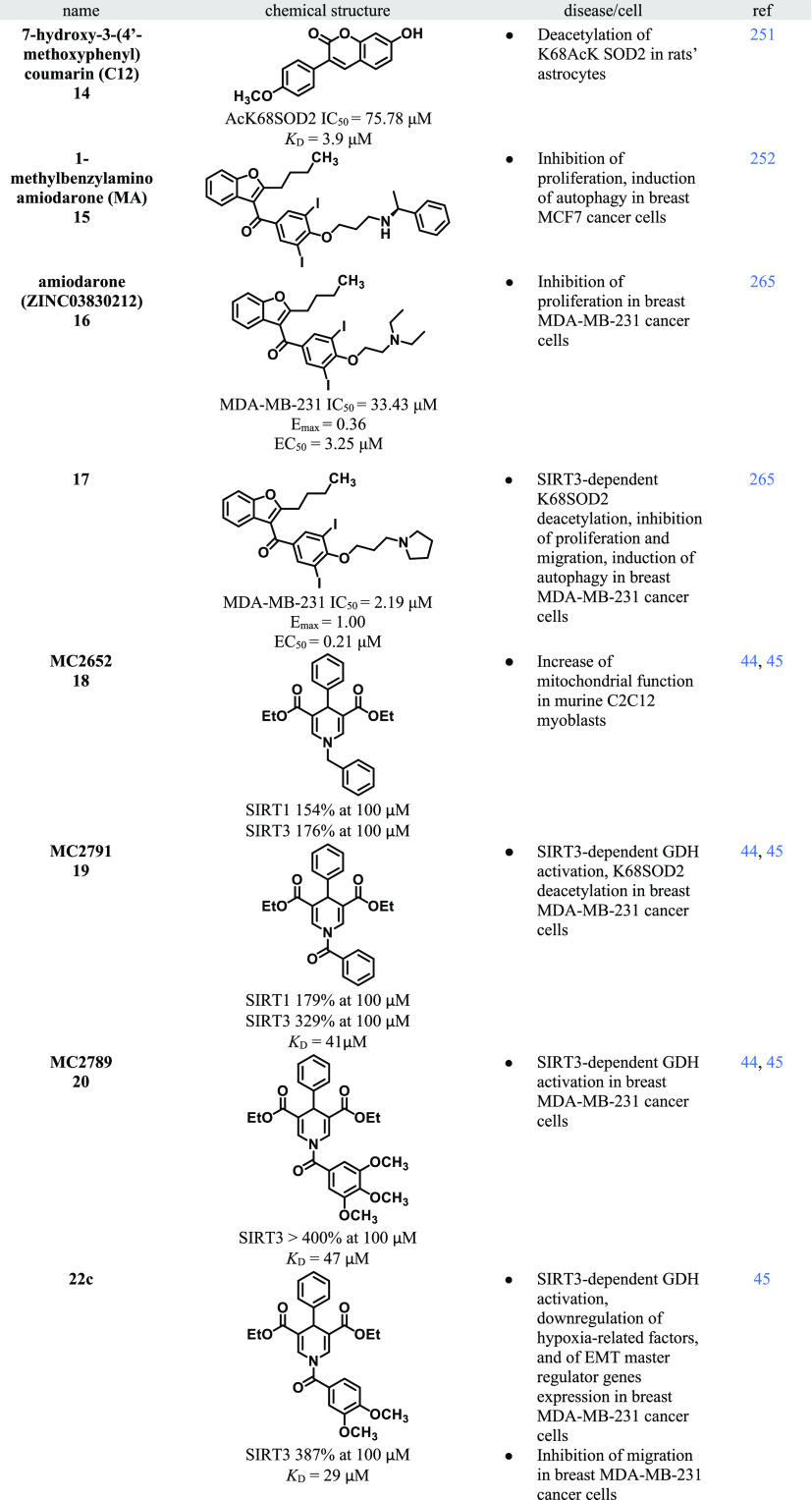

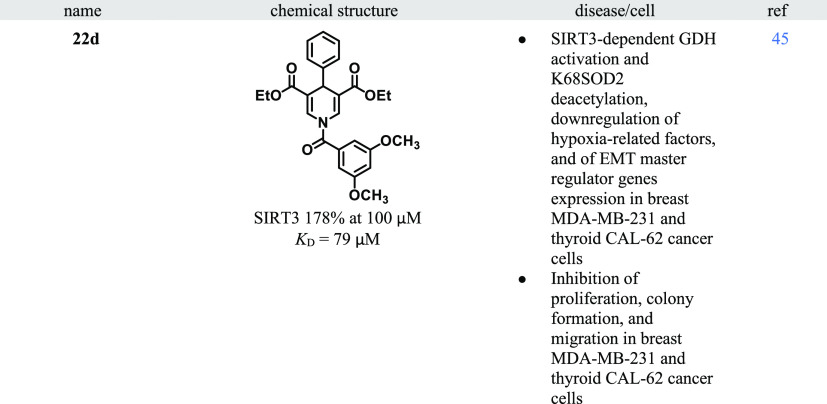
Summary of the SIRT3 Positive Modulators Discussed

**Figure 8:**
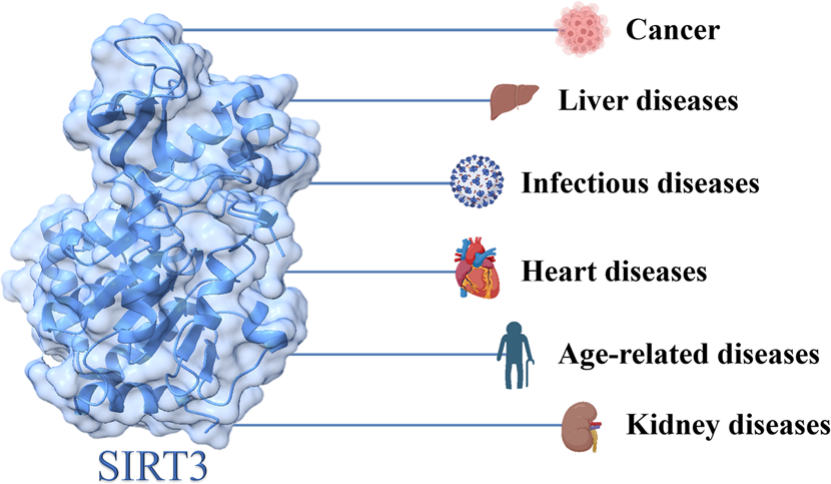
SIRT3 and human diseases.

The natural lignan honokiol (HKL) (**1**), extracted from the bark of magnolia, is a SIRT3 activator and was reported to ameliorate pre-existing cardiac hypertrophy in mice and to inhibit cardiac fibroblast proliferation and differentiation to myofibroblasts in a SIRT3-dependent AKT and extracellular signal-regulated kinases (ERK1/2) inhibition.^120^ The cardiotoxicity related to the antitumor agent doxorubicin is well-known.^191^ In a mouse model of doxorubicin-induced cardiomyopathy, **1** was able to activate SIRT3, promoting mitochondrial fusion and inhibiting apoptosis.^192^ Dynamin-related protein 1 (DRP1) is a protein involved in mitochondrial fragmentation and cell death, while Mfn1 and OPA1 are important players for outer and inner mitochondrial membrane fusion.^193^ Doxorubicin therapy reduces OPA1 and Mfn1 and increases DRP1, while under HKL therapy, these proteins return to normal levels.^194^ The process of mitochondrial fusion allows for the merging of mitochondrial contents, enhancing the ability of human cells to withstand high levels of detrimental mitochondrial DNA (mtDNA) damage (Figure 8).^193^ For this reason, **1** seems to rescue the healthy cardiac cells in tumor-xenograft mice while maintaining the effectiveness of doxorubicin as an antitumor treatment. Furthermore, **1** treatment can potentially block the proliferation of fibroblasts and their transition to myofibroblasts. Consequently, **1** can be used as an adjuvant in chemotherapy.^194^ Moreover, **1** stimulated SIRT3 activity, resulting in a blockage of the NF-κB-TGF-β1/Smad regulated inflammation and fibrosis signaling pathway in a renal fibrosis mouse model.^195^ A study by Ye et al.^196^ reported that by activating SIRT3 and consequently decreasing ROS and inhibiting apoptosis, compound **1** intervention could potentially enhance cognitive function in mice experiencing surgery- or anesthesia-related cognitive decline.^196^ Vitiligo is a condition for which some skin parts have no pigmentation. Activation of the SIRT3-OPA1 axis with **1** inhibited melanocyte apoptosis and ameliorated skin conditions.^174^

Another natural molecule with SIRT3 activating properties that could serve as an adjuvant in chemotherapy is Sylibin (**2**). **2** is a secondary metabolite derived from the seeds of blessed milk thistle (*Silybum marianum*), which is especially known for its antioxidant and anti-inflammatory properties in the liver.^197^**2** has been shown to improve mitochondrial function by controlling SIRT3 levels, resulting in a protective activity against cisplatin-induced AKI. A study by Li et al.^189^ reports that SIRT3 levels are lowered in tubular epithelial cells in a mouse model of cisplatin-induced acute kidney injury and that administration of **2** increased its expression, improving mitochondrial bioenergetics and kidney function. For this reason, **2** might be beneficial in the clinics in patients with cisplatin-induced AKI. Regrettably, the fundamental role that mitochondria play in safeguarding renal tubular epithelial cells during cisplatin-induced AKI is not yet fully understood.^189^

Osteoarthritis (OA) is a disease that affects a large part of the population.^198^ In this pathology, chondrocytes are degenerating due to several causes,^199,200^ such as mitochondrial dysfunction.^201,202^ Wang et al.^142^ demonstrated that SIRT3 very likely possesses protective properties in OA by maintaining mitochondrial homeostasis. They found significantly decreased levels of SIRT3 in degenerative knee articular cartilage and TNF-α treated chondrocytes. SIRT3 deficiency causes a decreased MMP, a reduced mitochondrial membrane permeability, and a reduced concentration of intracellular ATP, all being features of mitochondrial dysfunction.^142^ Dihydromyricetin (DHM, **3**), also known as ampelopsin, has anti-inflammatory, antioxidant, and antitumor activities.^203^ This compound exerts its neuroprotective effects under oxidative stress conditions via upregulating SIRT3 levels under hypobaric hypoxia.^204^ Considering the protective effects of compound **3** mediated by SIRT3 against chondrocyte degeneration, it is highly probable that compound **3** safeguards chondrocytes from degeneration by mitigating mitochondrial dysfunction. **3** seems to reduce oxidative stress by deacetylating K68 of SOD2 and thus promoting SOD2 enzymatic activity via SIRT3 activation in chondrocytes. Wang et al. reported that **3** was capable of upregulating the level of mitophagic markers in chondrocytes, indeed in chondrocytes treated with TNF-α **3** promotes the interaction between mitochondria and autophagosomes; thus, **3** can probably promote mitophagy via SIRT3 activation.^142^ Additionally, damaged mitochondria can be the prior source of ROS once they cannot be eliminated in time.^205^ To maintain mitochondrial homeostasis, mitophagy is mandatory. Moreover, in chondrocytes, SIRT3 can be positively regulated by **3** via the AMPK-PGC-1α-SIRT3 axis, knowing that PGC-1α can be an endogenous regulator of SIRT3.^142^

Polydatin (**4**), a combination of resveratrol and glucose, is a potent antioxidant derived from the root stem of *Polygonum cuspidatum*, a traditional Chinese herbal medicine,^206^ and has a longer half-life than resveratrol used alone.^207^ Many researchers claim that **4** can be used to treat oxidative stress-related diseases.^208,209^ Autophagy is an essential cellular process that maintains organelle function and protein quality.^210,211^ Zhang et al.^212^ demonstrate that pretreatment with **4** alleviated cardiac dysfunctions after myocardial infarction (MI) by increasing autophagy. **4** can improve cardiac function, decrease cardiomyocyte apoptosis, increase autophagy levels, and alleviate mitochondrial injury solely in the WT mice subjected to MI injury but not in the SIRT3^–/–^ ones. For these reasons, **4** seems to perform its effects against MI via SIRT3 activation.^212^

Pyrroloquinoline quinone (PQQ, **5**), an aromatic heterocyclic anionic orthoquinone, can be found in various edible plants. This molecule can neutralize superoxide and hydroxyl radicals, protecting mitochondria from oxidative stress.^213^ In HepG2 cells, compound **5** increases SIRT1 and SIRT3 levels, thus enhancing their mitochondrial function. It is worthwhile to investigate further the potential of **5** to serve as a therapeutic nutraceutical. Moreover, its chemical stability and solubility in water make **5** attractive as a therapeutic agent.^214^

Recently, caffeine (**6**) was also found to interact with SIRT3, displaying a *K*_D_ of 0.69 μM and promoting the binding of SIRT3 to its substrate. **6** enhanced the SOD2 activity through its lysines (K68 and K122) specific deacetylation and shielded skin cells from oxidative stress triggered by UV radiation both *in vitro* and *in vivo* model.^215^

Age-related macular degeneration (AMD) is a disease that leads to age-related irreversible vision loss. Pathogenesis of AMD is attributable to oxidative stress, autophagy, and apoptosis of retinal pigment epithelial (RPE) cells. The damage of RPE cells leads to foveal photoreceptor loss.^216^ Another natural compound, celastrol (**7**), is a pentacyclic triterpene compound derived from the *Tripterygium wilfordii* root bark.^217^ Previous studies report some anti-inflammatory and antioxidant activities for this compound.^218,219^ Du et al.^125^ show that **7** can have a protective effect on human ARPE-19 cells (a RPE cell line that spontaneously developed^220^) under H_2_O_2_-induced oxidative stress conditions, protecting them from apoptosis induction by activating the SIRT3 signaling pathway.^125^ When ARPE-19 cells are treated with **7**, the MTT assay shows an increase in survival in a dose- and time-dependent manner, decreasing oxidative stress. These effects could be ascribed to SIRT3 because there has been observed an increase in SIRT3 mRNA and protein expression levels in H_2_O_2_-induced ARPE-19 cells treated with **7**.^125^ Sesamin (**8**) is a lignin derived from sesame seeds belonging to the furo[3,4-*c*]furan class.^221^ Compound **8** ameliorates oxidative stress and death in brain injury models,^222^ improves cardiac function in doxorubicin-induced cardiotoxicity model,^223^ and attenuates nutritional fibrosing steatohepatitis.^224^ For these reasons, Fan et al.^225^ have studied the protective effects that **8** could have against cardiac remodeling. In an *in vivo* study, they found that **8** attenuates cardiac hypertrophy induced by an increase in pressure and fibrosis by upregulating SIRT3. The upregulation suppresses ROS production, which is one of the causes of cardiac fibrosis and vascular dysfunction processes underlying cardiac hypertrophy.^226^ In their study, they found that **8** abolishes TGF-β/Smad signaling in hypertrophied hearts, thus blocking cardiac fibrosis. Moreover, **8** normalizes inflammatory cytokines that promote the advancement of cardiac hypertrophy and heart failure. Using a selective SIRT3 inhibitor 3-TYP, **8**’s effects were blocked, suggesting that **8** acts by activating SIRT3 and thus by decreasing ROS, which are the key features of maladaptive cardiac remodeling.^225^ Oroxylin A (**9**) is a flavonoid derived from *Scutellaria baicalensis Georgi* root with anti-inflammatory,^227^ antiviral,^228^ and antitumor features.^91,229^ Wei et al.,^91^ in an *in vitro* study, found that **9** augments the expression and the relocation of SIRT3 in mitochondria. This results in the deacetylation of CypD and in the dissociation of hexokinase II (HK II), an enzyme involved in the glycolysis and in the regulation of the transcription from the mitochondria and thus in the glycolysis inhibition. HK II binds to the outer mitochondrial membrane protein voltage-dependent anion channel (VDAC) to mediate glycolysis of cancer cells,^230^ and CypD is mandatory to enhance the binding of HK II to VDAC. The HK II mitochondria binding is controlled by SIRT3, AKT, and GSK3β.^231,232^**9** seems to influence only SIRT3 and not the other two factors. Moreover, **9** seems to facilitate the SIRT3 relocation in mitochondria: remembering that SIRT3 translocates to the mitochondria after various stressors,^233^ this translocation could be linked with the boosted oxidative stress caused by **9**. Increased binding of HK II results in mitochondria-mediated apoptosis and can be linked to augmented cancer cell resistance to chemotherapeutic agents.^234,235^**9** might have a role in metabolic reprogramming by activating SIRT3.^91^ Fu et al.^171^ screened a library of 22 stilbene-based compounds, including resveratrol monomers, oligomers, and some semisynthetic derivatives, to develop novel efficacious neuroprotective agents able to activate SIRT3. They focused on HD, which is a hereditary neurodegenerative condition resulting from an anomalous expansion of polyglutamine in the protein known as Huntingtin (Htt).^236,237^ The pathogenesis of HD is not yet well understood, but it seems that energy metabolism, oxidative stress, excitotoxicity, and transcriptional dysregulation are involved.^238−241^ Several studies reported that mutant Htt-induced neurotoxicity is mediated by mitochondrial dysfunction.^242,243^ Because SIRT3 is implicated in the regulation of several mitochondrial enzymes, its activation could play a protective role in neurodegeneration. Moreover, in HD, aberrant Htt depletes SIRT3 protein levels. From the screening above mentioned, a stilbene resveratrol dimer with a five-member oxygen heterocyclic ring called *trans*-(−)-epsilon-viniferin (**10**) has been identified to increase SIRT3 protein levels. This increase might be due to enhanced protein translation or slowed down protein degradation, resulting in neuroprotection. **10** promotes mitochondrial biogenesis via SIRT3-driven activation of AMP-activated kinase and maintains mitochondrial function. Further, ROS production is associated with the pathogenesis of HD, and thus, SIRT3, through the induction of SOD2 activity, could reduce ROS levels.^171^

Recently, there has been a growing interest in drug repurposing toward the discovery of novel SIRT3 activators. Indeed, metformin (**11**), a well-known AMPK activator used as the initial treatment for individuals with type 2 diabetes, was shown to ameliorate type 2 diabetes-related atherosclerosis via SIRT3 upregulation.^244^

Adjudin (**12**) is a potent blocker of Cl^–^-channels extensively studied preclinically and clinically as a male contraceptive.^245^**12** safeguards cochlear hair cells in rodents from gentamicin-induced hearing damage through the SIRT3–ROS pathway, both in laboratory experiments and in living subjects.^246^

Melatonin (**13**), a hormone derived from serotonin and produced in the pineal gland, has been discovered to boost the expression of SIRT3 and serves as a protective factor in the context of heart disease,^247^ liver injury,^248^ and atherosclerosis.^4,249^ Furthermore, in an atherosclerosis mouse model, **13** proved to activate SIRT3–FOXO3a–Parkin regulated mitophagy, thus preventing inflammation and atherosclerotic progression.^4,249^**13** upregulated the SIRT3 expression after transient middle cerebral artery occlusion, thus ameliorating cerebral ischemia/reperfusion injury.^250^ An *in vivo* study reports that **13** can ameliorate mitochondrial oxidative damage apoptosis and can preserve mitochondrial function in the aged ovarian. In particular, **13** seems to enhance SIRT3 activity and FOXO3a nuclear translocation, which leads to transactivation of the antioxidant genes, resulting in the inhibition of mitochondrial oxidative damage.^126^ As already mentioned above, SOD2 is inhibited by constitutive acetylation, and SIRT3 can modulate its activity.

Lu et al.^251^ resolved the first crystal structure of SOD2K68AcK. Based on the latter, they built a compound-screening model by using the SIRT3–SOD2K68AcK reaction system. A screening of a compound library using this reaction system allowed to disclose 7-hydroxy-3-(4′-methoxyphenyl)coumarin (C12, **14**) able to promote SOD2K68 deacetylation by binding the SIRT3 complex. Through ITC assay, the authors found that **14** displayed a *K*_D_ of 3.9 μM. Moreover, they also found the SOD2K68AcK deacetylation IC_50_ value of 75.78 μM.^251^ By docking study, these researchers studied the **14** binding mode to the SOD2K68AcK–SIRT3 complex, and they discovered that **14** can form H-bonds with Ser253 and Asn229. These interactions allow the SIRT3 conformational change and, therefore, the contact of AcK68 with the NAD^+^ binding pocket of SIRT3. Finally, **14** was able to reduce superoxide levels within the cell by directly activating endogenous SIRT3. However, a very high concentration (300 μM) of **14** was needed to activate endogenous SIRT3.^251^

In 2021, Zhang et al.^252^ studied the role of SIRT3 in autophagy. They found that overexpression of SIRT3 could stimulate autophagy and then the cellular homeostatic mechanism. Primarily, they found out that the overexpression of SIRT3 may be a promising therapeutic strategy for the treatment of invasive breast cancer in which autophagy is known to be deregulated.^253,254^ Through a multiple docking strategy applied to 212, 255 compounds followed by a structure-based molecular docking, the authors discovered a new SIRT3 activator, 1-methylbenzylamino amiodarone (MA) (**15**). The *in silico* data are further supported by the results of autophagic and SIRT3 activation experiments. In MCF-7 cells, **15** lowered the acetylation levels on K68 and K122 of SOD2 and was able to increase the expression of some genes that promote autophagy, such as ATG4B, ATG5, and beclin-1. Moreover, **15** suppressed the migration of MCF-7 cells by increasing the E-cadherin expression. Finally, the researchers decided to extend their studies on **15** with *in vivo* experiments using a xenograft MCF-7 breast cancer model, confirming the *in vitro* data. Furthermore, the reduction of *K*i-67 expression as a result of **15** treatment proved its capability to hamper tumor growth.^252^

The mentioned compounds (**1**–**13**) include natural compounds or those derived from drug repurposing or in the case of **14** and **15** from a focused virtual screening approach. For several of these compounds (**2**, **6**, **10**, **12**, **14**, and **15**), no data regarding their activity on other sirtuin isoforms could be found in the literature. The other mentioned compounds are often not selectively activating SIRT3. Specifically, natural compounds **1**,^255^**3**,^256−258^**4**,^259,260^**5**,^214^**8**,^223,261^**9**,^262^ and drug **11**,^263^ demonstrate the capability to also activate SIRT1. Compound **7** exhibits the direct induction of the SIRT7 gene expression, whereas compound **13** elicits the expression of SIRT1, SIRT6, and SIRT7.^264^ However, all these compounds (**1**–**15**) could be considered potential lead compounds to be optimized through a med-chem approach.

The following compounds presented herein underwent, after their hit discovery, various medicinal chemistry optimizations, which led to highly selective SIRT3 activators.

**Figure 9:**
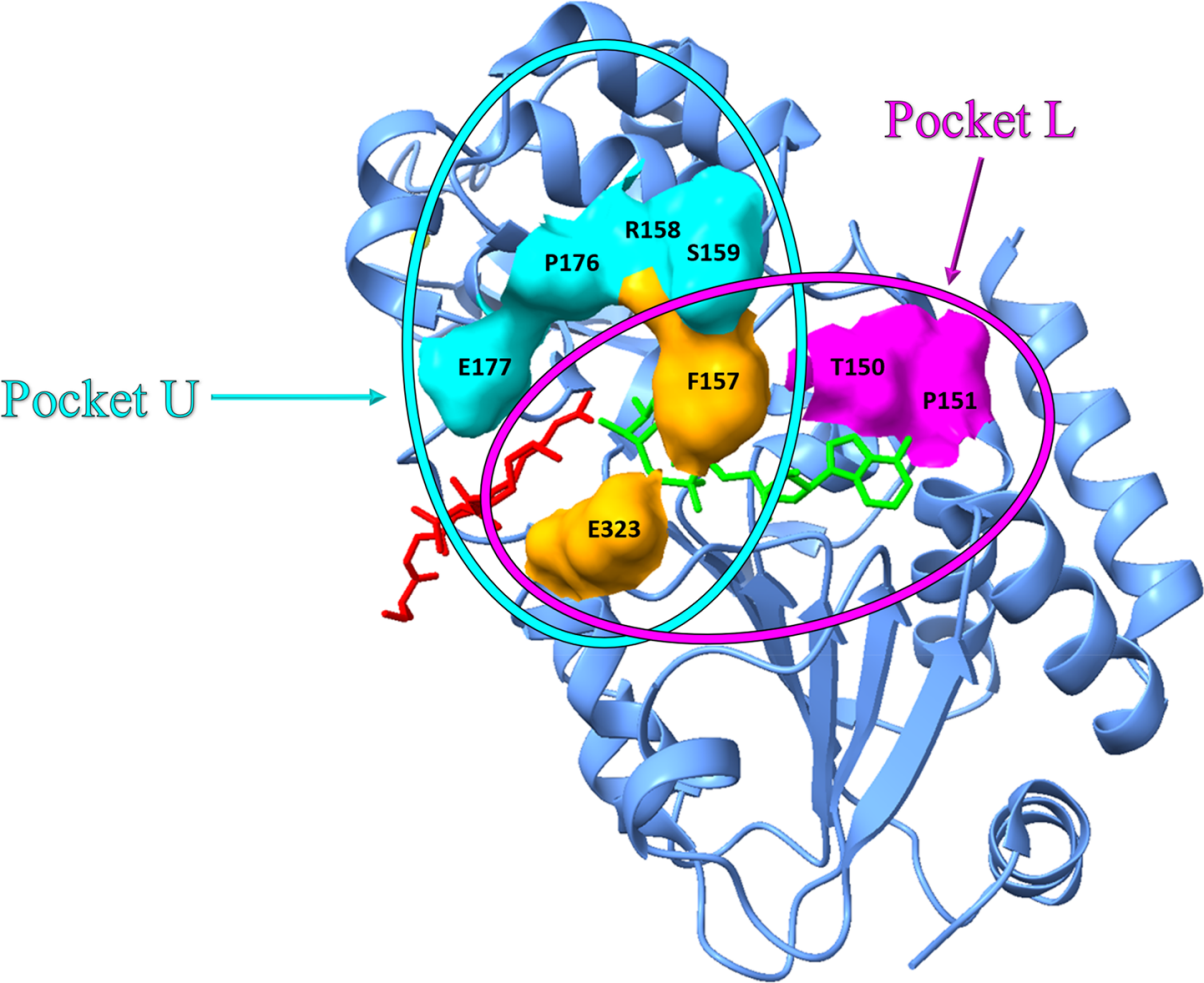
Crystal structure of SIRT3 in complex with carbaNAD (in green) and acetylated ACS2 peptide (in red). Pocket U is depicted in cyan, while pocket L is depicted in magenta with a surface style. F157 and E323 are residues in common with the two pockets and are depicted in orange (PDB 4FVT).

**Figure 10:**
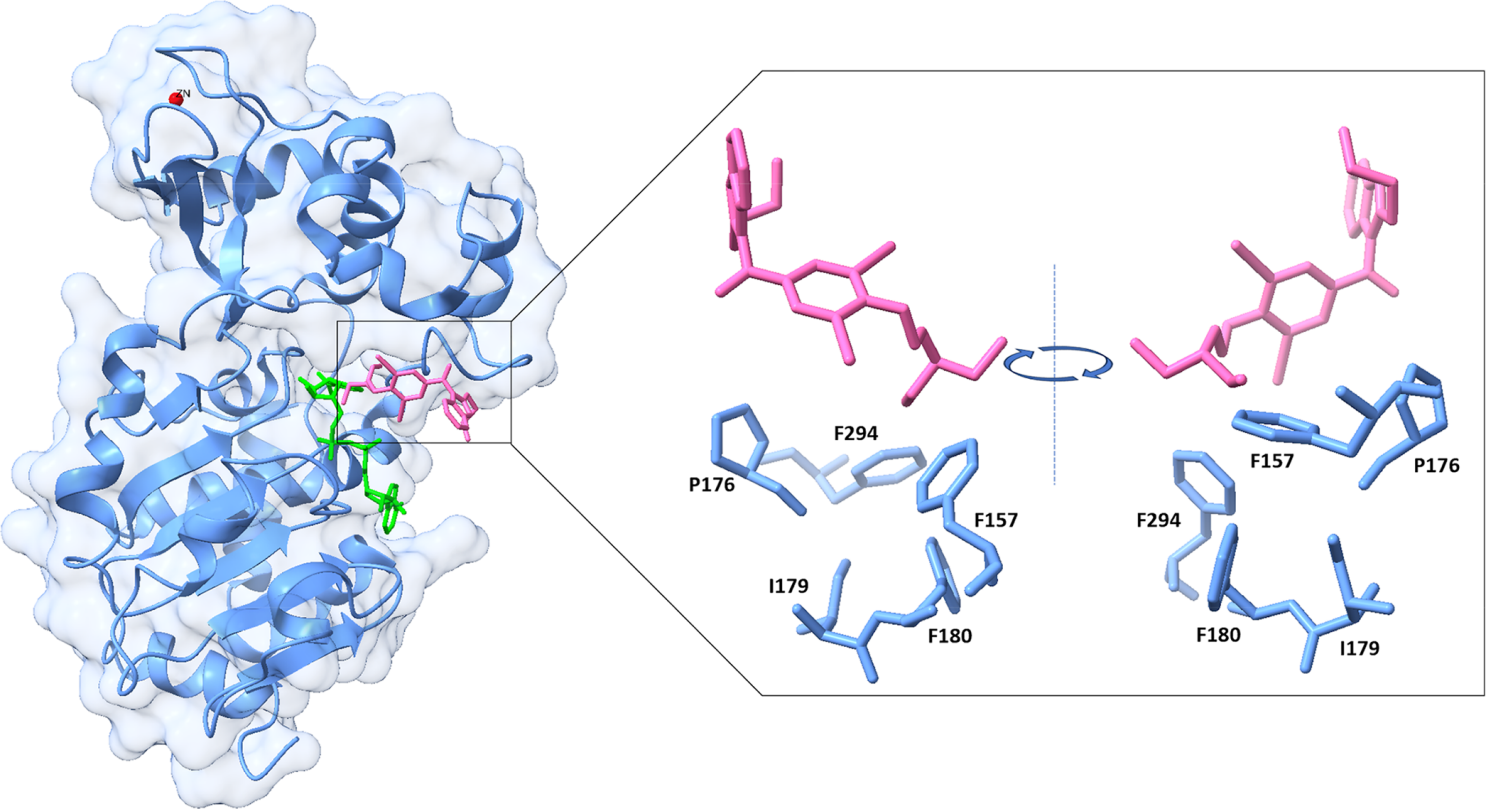
Focus on the residues of SIRT3 with which the diethylamino group of **16** interacts. Amiodarone (**16**) is depicted in pink, while NAD^+^ is depicted in green (PDB 5H4D).

**Figure 11:**
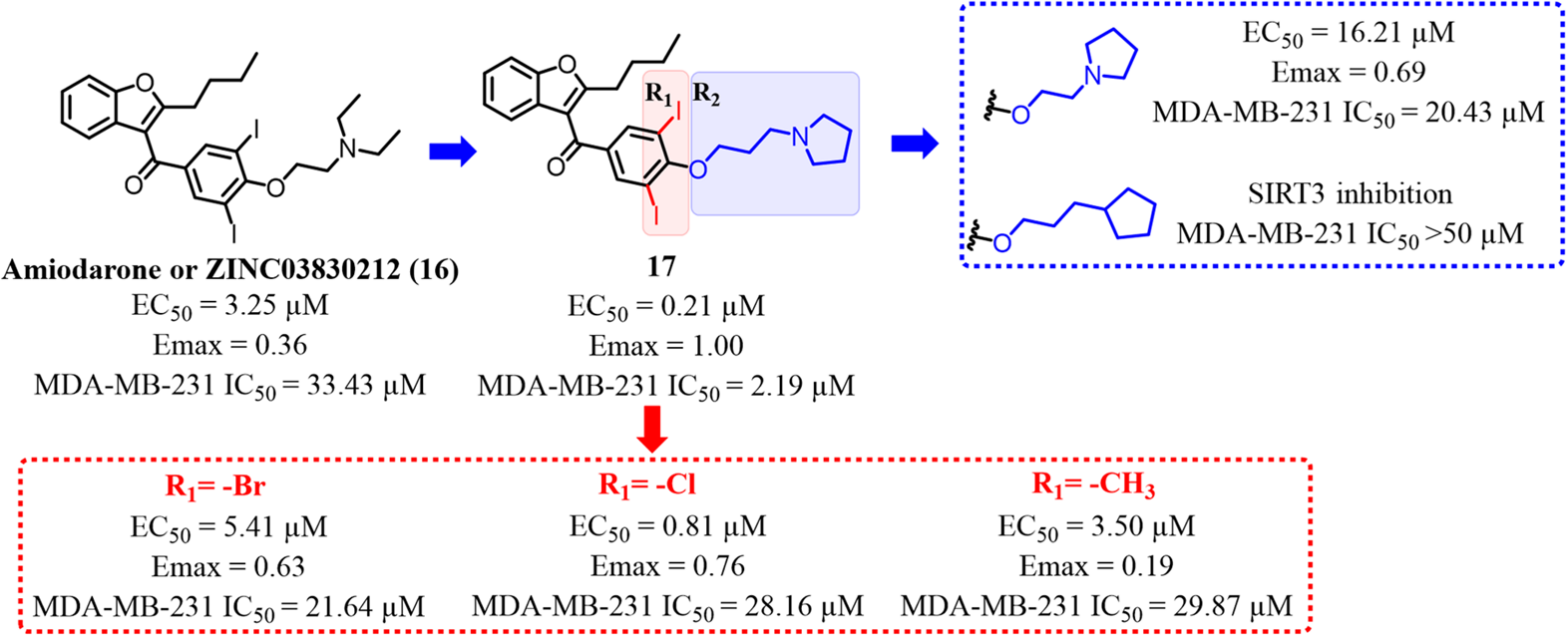
Development and main SAR study of **17**.

To discover molecules that specifically modulate SIRT3, it is necessary to find differences in the sirtuins family structure. In particular, SIRT3 seems to have two important pockets: L and U. These two pockets could allow it to achieve a specific binding for SIRT3. Pocket L is composed of T150, P151, F157, and E323, while pocket U is formed by F157, R158, S159, P176, E177, and E323. F157, R158, and S159 that are near the flexible region of SIRT3, allowing the NAD^+^ binding (Figure 9). Consequently, small molecules targeting pocket L or U could allosterically regulate SIRT3. Through a screening of 1.4 million small molecules against pocket U, Zhang et al. found that compound ZINC03830212 (**16**), corresponding to amiodarone, a well-known class III antiarrhythmic agent, blocking the myocardial potassium channels in cardiac tissue, displayed an activation EC_50_ of 3.25 μM toward SIRT3. The cocrystal structure of **16** in complex with SIRT3 was solved. The diethylamine tail interacts with F157, I179, P176, F180, and F294, while the benzofuran core functions as a support (Figure 10). Further, in the same study and through a combined structure-guided design and high-throughput screening approach, Zhang et al. have also disclosed a small molecule as a specific SIRT3 activator, compound **33c** (**17**), by modifying the structure of compound **16**.^265^ Compound **17** differs from **16** for the substituent at the 4 position of the benzoyl group: **16** has a 2-(diethylamino)ethoxylic group, while **17** replaces it with a 3-(pyrrolidin-1-yl)propoxy)phenylic group. Compound **17** was able to inhibit the proliferation and migration of human MDA-MB-231 triple-negative breast cancer (TNBC) cells via SIRT3-driven autophagy/mitophagy signaling pathways *in vitro* and *in vivo*. **17** has an IC_50_ in MDA-MB-231 cells of 2.19 ± 0.16 μM, *E*max and EC_50_, respectively, are 1.00 ± 0.07 and 0.21 μM. Compound **17** was more selective for SIRT3 with respect to SIRT1, SIRT2, and SIRT5 due to the interaction with F157, R158, and F294. For the *in vivo* studies, an MDA-MB-231 TNBC xenograft model was used. **17** shows an antiproliferative activity in a dose-dependent (25, 50, and 100 mg/kg) manner after 16 days of treatment. **16** possesses a well-known lung toxicity.^266^ Given the similarity of **17** to amiodarone structure, the authors studied its pulmonary toxicity in the same MDA-MB-231 TNBC xenograft model. In the high-dose group, **17** shows a certain level of toxicity associated with the expansion of the pulmonary septum. The study also reports a SAR analysis in which the authors changed the halogen atom and the side chain of **17**, but none of the chemical modifications improved the biochemical activity toward SIRT3 (Figure 11).^265^

**Figure 12:**
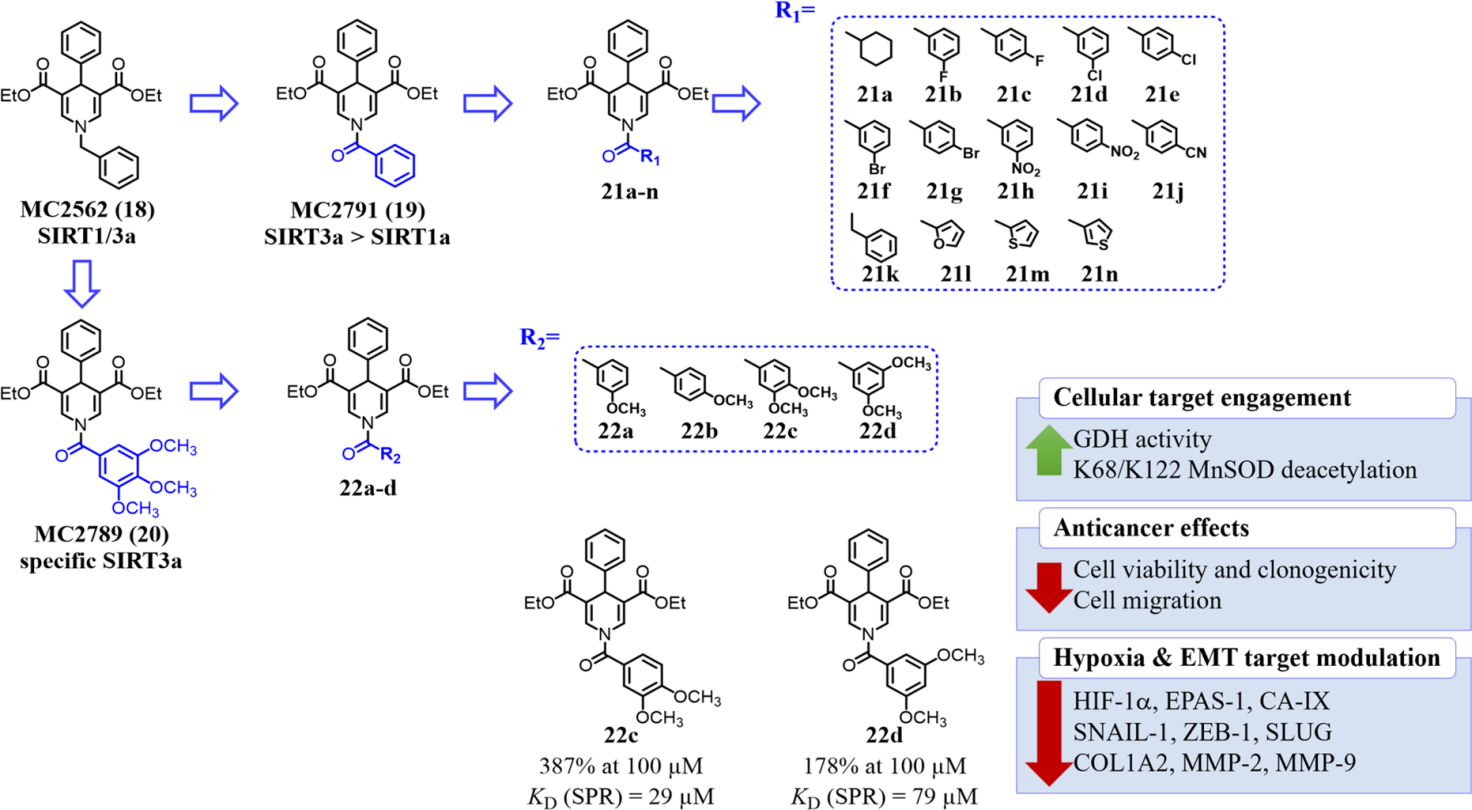
Development of novel 1,4-DHP-containing SIRT3 specific activators.

Also, our research group has been working for almost 15 years on the discovery and development of SIRTs activators. Indeed, our group described the 1,4-dihydropyridine (1,4-DHP) nucleus as potent SIRTs activators^267−269^ and very recently reported the identification and characterization of 1,4-DHP-based compounds as specific activators of SIRT3 or SIRT5 (Figure 12).^44^ To avoid artifacts and other nonspecific effects, the SIRT3 or SIRT5 activation by the most potent compounds was confirmed through a PncA/GDH-coupled deacylation assay instead of the “Fluor-de-Lys” (FdL) substrate; moreover, the direct SIRT activation was also validated by MS-based experiments and proved in cellular settings. The development of the SIRT3-specific compounds started from MC2562 (**18**), an activator of both SIRT1 and SIRT3, on whose structure we added a carbonyl group at the methylene between the nitrogen atom of the 1,4-DHP and the phenyl ring, thus obtaining MC2791 (**19**), an activator of SIRT3 (over 300% at 100 μM) and to a lesser extent SIRT1 (below 200% at 100 μM). Next, by replacing the benzoyl moiety at the N1 position with 3,4,5-trimethoxy benzoyl group, we obtained the compound MC2789 (**20**), a very strong SIRT3-specific activator (over 400% at 100 μM). In triple-negative MDA-MB-231 breast cancer cells, **19** and **20** increased GDH activity, a known SIRT3 substrate activated by deacetylation, similarly or better than the effect showed by overexpressing SIRT3 cells. In addition, western blot analysis proved the AcK68SOD2 deacetylation effect by compound **19** treatment.^44^

In a follow-up paper,^45^ our group designed a novel small series of **19** analogues by adding several functional groups at the *meta* or *para* position of the phenyl ring or by substituting the phenyl ring with some bioisosteres or saturated analogues or by deleting the carbonyl group between the phenyl ring and the 1,4-DHP. Furthermore, starting from **20**, also analogues of this compound were obtained by eliminating the methoxy groups on the 3,4,5-trimethoxybenzoyl moiety one by one (Figure 12). Among the novel compounds coming from the deletion of the methoxy groups, the 3,4-dimethoxy substituted **22c** was the strongest SIRT3 activator in biochemical activation assays. **22c** stimulated SIRT3 activity of 387% at 100 μM and displayed a *K* of 29 μM (SPR assay). When tested for assessing the target engagement in MDA-MB-231, it emerged **22d** with 175% GDH activation, comparable to the reference compound **20** and more effective than the SIRT3-overexpression. Moreover, **22d** showed the highest time-dependent activity in reducing the cell viability and colony formation in both normoxia and hypoxia environments when tested on MDA-MB-231 and thyroid CAL-62 cancer cells. Particularly, **22d** displayed IC_50_ values of 2.5–16.8 μM (normoxia) and 3.5–5.4 μM (hypoxia) in CAL-62 cells, 2.2–3.6 μM (normoxia), and 3.2–10.6 μM (hypoxia) in MDA-MB-231 cells. **22d** also showed a significant downregulation of HIF-1α, EPAS-1 (HIF-2α), and carbonic anhydrase IX (CA-IX), all proteins involved in several hallmarks of cancer. By evaluating the EMT master regulator genes’ expression, it was found that **22c**, and especially **22d**, lowered SNAIL, ZEB1, COL1A2, MMP2, and MMP9 genes expression after 48 h of treatment at 50 μM. SLUG gene expression was diminished only in hypoxia conditions. Generally, the effect of the two compounds was clearly stronger in hypoxic conditions, highlighting the function of SIRT3 in the modulation of hypoxia-dependent gene expression. Of note, **22c** and, to a better extent, **22d** reduced the MDA-MB-231 cell migration after 48h of treatment at 50 μM.^45^

## Perspectives

SIRT3 is a mitochondrial sirtuin able to regulate several metabolic pathways such as OXPHOS, TCA, and urea cycle, amino acid metabolism, fatty acid oxidation, mitochondrial dynamics, and the mitochondrial unfolded protein response (UPR).^4,23,24^ The most exciting role of SIRT3 lies in its ROS scavenging capabilities because the accumulation of ROS causes several health problems. Given the involvement of SIRT3 in these important pathways, its deregulation can result in several human diseases, such as cancer^73^ as well as noncancer pathologies such as liver,^110,111,189^ heart,^77,115,124^ metabolic,^43,108,137,139,186^ age-related,^125,126^ kidney,^123,133−137^ neurodegenerative^162^ diseases, neuropathic pain,^152^ osteoarthritis,^142^ and also infective disease caused by HBV^112^ and COVID-19.^113^

In the present perspective, we have updated the SIRT3 biological functions and several positive modulators of SIRT3. The latter include several natural compounds (**1**–**10**), examples of molecules of pharmaceutical interest (**11**,**12**), and even a hormone (**13**) compound. Among these, compound **1** stands out as the most potent natural activator of SIRT3; indeed, **1** activates SIRT3 and offers a range of therapeutic benefits. It ameliorates cardiac hypertrophy and inhibits fibroblast proliferation in a SIRT3-dependent manner^120^ but also protects against doxorubicin-induced cardiomyopathy by promoting mitochondrial fusion and inhibiting apoptosis via SIRT3 activation.^192^ It also exhibits anti-inflammatory and antifibrotic effects in renal fibrosis.^195^ Moreover, **1** may enhance cognitive function^196^ and improve vitiligo by activating the SIRT3 pathway.^174^ Most SIRT3 activators from natural origin or repurposed drugs such as compounds **1**, **3**, **4**, **5**, **7**, **8**, **9**, **11**, **13**, **14**, and **15** are not selective for SIRT3, while for the compounds **1**, **2**, **6**, and **10** no data are yet available in the literature. From this, it can be stressed that the great limitation of natural compounds and of those deriving from drug repurposing is the lack of selectivity. However, these compounds merit further studies as they present promising opportunities as potential hit candidates for a med-chem optimization.

Recently, Lu et al.^251^ built a compound-screening model using the SIRT3–SOD2K68AcK reaction system used to discover **14**. In 2021, Zhang et al.^252^ identified **15** that impedes breast tumor growth both *in vitro* and *in vivo* via activation of SIRT3.^252^

After a screening involving 1.4 million compounds by Zhang et al.,^265^ compound **16** was identified with a single-digit micromolar EC_50_. After a structure–activity relationship investigation starting from **16**, the researchers discovered the above-mentioned compound **17** with a submicromolar EC_50_ value. **17** inhibits the proliferation and the migration of human MDA-MB-231 cells via SIRT3-driven autophagy/mitophagy signaling pathways both *in vitro* and *in vivo*. However, **17** displays some lung toxicity when tested in an MDA-MB-231 TNBC xenograft model. Of note **17** was not tested for off-target effects, i.e., its antiarrhythmic effect, considering that **16** is an approved drug for that. This point is crucial in the process of the med-chem optimization of repurposed drugs, as the original targets should no longer be affected.

Based on our previous studies in which 1,4-DHP-based compounds as specific activators of SIRTs were discovered and developed,^44,267−269^ compounds **22c** and **22d** were developed. The two compounds display an activation of SIRT3 greater than 150% and a good binding affinity for the enzyme. Notably, **22d** shows reduced cell viability and colony formation in normoxia and hypoxia environments when tested on breast MDA-MB-231 and thyroid CAL-62 cancer cells with single-digit micromolar IC_50_ values. **22d** is also able to downregulate hypoxia-induced factors (HIF-1α, EPAS-1, and CA-IX) and epithelial-mesenchymal transition master regulators and extracellular matrix components (SNAIL1, ZEB1, SLUG, COL1A2, MMP2, and MMP9), other than impairing MDA-MB-231 cell migration.^45^

Most SIRT3 positive modulators described so far are rather unspecific pleiotropic natural compounds or repurposed drugs not yet optimized for the new target, thus their application in preclinical or even clinical settings is rather limited. Despite numerous efforts over the past decade to develop positive modulators, very few compounds can be considered as promising leads toward potent and selective activators. As summarized in Table 1, SIRT3 is involved in numerous diseases, but the lack of proper tools limited the possibility to study small molecule activation in these pathologies.

The problem is related to a lack of structural information on how to develop a SIRT3 activator. The design of inhibitors is easier for medicinal chemists as they are mainly trained for inhibition, as most targets require this. Furthermore, for a more comprehensive understanding of enzyme activation it would be useful to consider the application of enzyme kinetic models, in addition to the activation percentage. Indeed, as outlined in foundational textbooks, such as that authored by Leskovac,^270^ enzyme activation can be systematically quantified using specific kinetic models, resulting in a more objective and universally applicable description of the activation mechanism of a compound. It would be helpful to evaluate some kinetic parameters such as the β parameter that measures the magnitude of the increase of the catalytic reaction constant as well as the activator binding constant.^271^ The development of activators is even more challenging because the interaction mechanism between SIRT3 and agonists is not yet known. Therefore, hopefully, in the future, with the help of technologies/methodologies such as fluoHTS or/and DNA encoded library and more useful information about the binding mechanism of activators, it will be possible to develop molecules with high potency and specificity worthy of therapeutic application. As our research team contribution, the aim will be addressed to optimization of our 1,4-DHP scaffold, with the hope of obtaining a cocrystal structure between our best molecules and SIRT3 in order to continue the SAR investigation with a structure-based approach and to obtain more potent and selective SIRT3 activators. In the years to come, we can expect novel potent activators and optimization of the currently known hit compounds, which can then be studied and hopefully applied in various disease models *in vitro* and *in vivo*.

## Abbreviations Used

1,4-DHP: 1,4-dihydropyridine
3-TYP: 3-(1*H*-1,2,3-triazol-4-yl) pyridine
4-HNE: 4-hydroxynonenal
ACC1: acetyl-coA carboxylase 1
AceCS2: acetyl-CoA synthase 2
AKI: acute kidney injury
NAFLD: nonalcoholic fatty liver disease
AKT: protein kinase B
AMD: age-related macular degeneration
AMPK: AMP-activated protein kinase
Ang-2: angiopoietin-2
ARNT: aryl hydrocarbon receptor nuclear translocator
ATG5: **7**
4B: autophagy related 5, 7, 4B
CA-IX: carbonic anhydrase IX
cccDNA: covalently closed circular DNA
CKD: chronic kidney diseases
CLL: chronic lymphocytic leukemia
COL1A2: collagen type I alpha 2 chain
CR: caloric restriction
CypD: cyclophilin D
DHM: dihydromyricetin
DRP1: dynamin-related protein 1
DRP1: dynamin-related protein1
ECM: extracellular matrix
EMT: epithelial–mesenchymal transition
ETC: electron transport chain
FAK: focal adhesion kinase
FLSIRT3: full length SIRT3
GAPDH: glyceraldehyde-3-*P*-dehydrogenase
GBC: gallbladder cancer
GDH: glutamate dehydrogenase
GDH: glutamate dehydrogenase
GLUT1: glucose transporter 1
GOT: glutamate oxaloacetate transaminase
GOT2: glutamate oxaloacetate transaminase 2
GPX: glutathione peroxidase
GSH: reduced glutathione
GSK3β: glycogen synthase kinase 3β
GSSG: oxidized glutathione
HBV: hepatitis B virus
HCC: hepatocellular carcinoma
HD: Huntington’s disease
HFD: high fat diet
HIF1α: hypoxia-inducible factor-1a
HIFs: hypoxia-inducible factors
HK II: hexokinase II
VDAC: voltage-dependent anion channel
HKL: Honokiol
FOXO: forkhead box O
HMGCS2: 3-hydroxy-3-methylglutaryl CoA synthase 2
HNSCC: head and neck sqamous cell carcinoma
HO-1: hemoxygenase 1
HRE: HIF response element
HSP90: heat shock protein 90
Htt: Huntingtin
IDH2: isocitrate dehydrogenase 2
ISCU: iron–sulfur cluster assembly protein
ITC: isothermal titration calorimetry
JNK: Jun N-terminal kinase
KO: knockout
LCAD: long-chain acyl CoA dehydrogenase
LKB1: liver kinase B1
LncRNAs: long noncoding RNA
MA: 1-methylbenzylamino amiodarone
MAFLD: metabolic dysfunction-associated fatty liver disease
MDA: malondialdehyde
MEFs: mouse embryonic fibroblasts
Mfn2: mitofusin-2
ERK1/2: extracellular signal-regulated kinases
mHtt: mutant huntingtin
MI: myocardial infarction
miRNA: microRNA
MMP: mitochondrial membrane potential
MPP: matrix processing peptidase
MPTP: 1-methyl-4-phenyl-1,2,3,6-tetrahydropyridine
mPTP: mitochondrial permeability transition pore
mtDNA: mitochondrial DNA
mTOR: mammalian target of rapamycin
MTT: 3-(4,5-dimethylthiazol-2-yl)-2,5-diphenyl-2*H*-tetrazolium bromide
NF-kB: nuclear factor kappa B
NMNAT2: nicotinamide mononucleotide adenylyltransferase 2
NMNAT3: nicotinamide mononucleotide adenylyltransferase 3
NRF2: erythroid 2-related factor
O-AADPR: *O*-acyl ADP-ribose
OA: osteoarthritis
OGG1: 8-oxoguanine-DNA glycosylase 1
OPA1: optic atrophy 1
OTC: ornithine transcarbamoylase
OXPHOS: oxidative phosphorylation
PARP1: poly ADP-ribose polymerase 1
PD: Parkinson’s disease
PDAC: pancreatic ductal adenocarcinoma
PDC: pyruvate dehydrogenase complex
PDH: pyruvate dehydrogenase
PGC-1α: PPARγ coactivator 1
PHD: prolyl hydroxylase domain
PINK1: PTEN-induced kinase1, phosphatase and tensin homologue-induced kinase 1
PQQ: pyrroloquinoline quinone
PYCR1: pyrroline-5-carboxylate reductase 1
RIP: receptor-interacting protein
ROS: reactive oxygen species
RPE cells: retinal pigmenti epithelial cells
SDH: succinate dehydrogenase
SENP1: sentrin-specific protease 1
SETD1A: SET domain containing protein 1A
SHMT2: serine hydroxymethyltransferase 2
SIRT3: sirtuin 3
SNAI1: snail family transcriptional repressor 1
SNI: spared nerve injury
SOD2: manganous superoxide dismutase 2
STAT3: signal transducer and activator of transcription 3
*t*-BOOH: *tert*-butyl hydroperoxide
TBI: traumatic brain injury
TCA: tricarboxylic acid
TGF-α: transforming growth factor α
TGF-β: transforming growth factor β
TNBC: triple negative breast cancer
UBF: upstream binding factor
UCP2: mitochondrial uncoupling protein 2
ULK1: Unc-51-like autophagy-activating kinases 1
UPR: unfolded protein response
VEGF: vascular endothelial growth factor
VLDL: very low-density lipoprotein
WT: wild-type
ZEB1: zinc finger E-box-binding homeobox 1.
